# A Novel 2006 Indian Outbreak Strain of Chikungunya Virus Exhibits Different Pattern of Infection as Compared to Prototype Strain

**DOI:** 10.1371/journal.pone.0085714

**Published:** 2014-01-20

**Authors:** Abhishek Kumar, Prabhudutta Mamidi, Indrani Das, Tapas K. Nayak, Sameer Kumar, Jagamohan Chhatai, Subhasis Chattopadhyay, Amol R. Suryawanshi, Soma Chattopadhyay

**Affiliations:** 1 Department of Infectious Disease Biology, Institute of Life Sciences, Bhubaneswar, Odisha, India; 2 School of Biological Sciences, National Institute of Science Education & Research, Bhubaneswar, Odisha, India; Emory University School of Medicine, United States of America

## Abstract

**Background:**

The recent re-emergence of Chikungunya virus (CHIKV) in India after 32 years and its worldwide epidemics with unprecedented magnitude raised a great public health concern.

**Methods and Findings:**

In this study, a biological comparison was carried out between a novel 2006 Indian CHIKV outbreak strain, DRDE-06 and the prototype strain S-27 in mammalian cells in order to understand their differential infection pattern. Results showed that S-27 produced maximum number of progenies (2.43E+06 PFU/ml) at 20 to 24 hours post infection whereas DRDE-06 produced more than double number of progenies around 8 hours post infection in mammalian cells. Moreover, the observation of cytopathic effect, detection of viral proteins and viral proliferation assay confirmed the remarkably faster and significantly higher replication efficiency of DRDE-06. Moreover, our mutational analysis of whole genome of DRDE-06 revealed the presence of nineteen mutations as compared to S-27, whereas the analysis of 273 global isolates showed the consistent presence of fifteen out of nineteen mutations in almost all outbreak isolates. Further analysis revealed that ∼46% of recent outbreak strains including DRDE-06 do not contain the E1-A226V mutation which was earlier shown to be associated with the adaptation of CHIKV in a new vector species, *Aedes albopictus*.

**Conclusions:**

A novel 2006 Indian CHIKV outbreak strain, DRDE-06 exhibits different pattern of infection as compared to prototype strain, S-27. This might be associated to some specific mutations observed in genome wide mutational analysis in DRDE-06 which emphasizes the need of future experimental investigation.

## Introduction

Chikungunya fever is an acute mosquito-borne febrile arthritis caused by *Chikungunya virus* (CHIKV), an *Alphavirus* belonging to *Togaviridae* family [1]. The disease is characterized by abrupt onset of high fever, arthralgia, myalgia, headache, rash [2]–[5] and poly-arthralgia which is very painful and may persist for several months in some cases [6]. CHIKV is an enveloped virus comprising of 11.8 kb long positive sense single stranded RNA genome. The 5′ end ORF encodes for four non-structural proteins, nsP1-4, known to be involved in viral replication and the 3′ end ORF encodes for three major structural proteins, capsid, E1, and E2 [1], [7], [8].

This virus was first isolated in Tanzania, Africa in 1952 [9] and in last 60 years, several CHIKV outbreaks have occurred globally [5], [10]–[12]. However, extensive CHIKV outbreak in 2005–2007 in the Indian ocean island followed by subsequent outbreaks in different parts of Asia including India, Indonesia, Malaysia, Sri Lanka, Thailand, New Guinea, China [10]–[16] have raised a major public health concern in many countries of the world.

In India, the CHIKV outbreak was first recorded in Kolkata in 1963 [5], [12] and was followed by epidemics in Chennai, Pondicherry, and Vellore in 1964; Visakhapatnam, Rajamundry, Kakinada and Nagpur in 1965 and at Barsi in 1973 [5], [12]. After a gap of 32 years, CHIKV infection has reemerged in the form of recent outbreaks in India during 2005–08 affecting 1.3 million people in 13 states [12]. The clinical manifestations during these outbreaks are found to be more severe compared to the classical cases [17] which lead to the speculation that either a more virulent or an efficiently transmitted variant of this virus may have emerged in recent years. Based on CHIKV E1 sequences, there are three different groups of CHIKV strains viz. East Central South African (ECSA), West African and Asian. It has been observed that the recent outbreaks from 2005 onwards are caused by ECSA type of CHIKV strains. The CHIKV prototype strain S-27 which was isolated in 1952 in Tanzania, Africa and the recent outbreak strain DRDE-06 which has been isolated in 2006 from Southern India during outbreak 2005 to 2008, both belong to the ECSA type [17], [18]. Hence, an attempt has been made to investigate the differences in biological phenotypes of these two viruses, if any, and to understand the possible explanation of its epidemic emergence.

In the present study, we investigate the infection pattern and biological properties of two Chikungunya strains, one is the prototype strain S-27 and another is a novel 2006 Indian outbreak strain, DRDE-06 [17], [18] in order to understand whether a highly infective variant of this virus has emerged in the recent years. This has been performed by estimating cytopathic effect (CPE), viral protein expression and viral particle formation after infecting mammalian cells. Moreover, mutational analysis of whole genome sequences of 273 global CHIKV isolates has been carried out with reference to S-27 and DRDE-06 to provide probable explanation of our observations and also to elucidate the reasons of the recent global epidemics.

## Materials and Methods

### Cells, Viruses and Antibodies

Vero cells (African green monkey kidney fibroblasts), C6/36 (*Aedes albopictus* mosquito larva cells), Chikungunya virus strains S-27 (accession no. AF369024.2) and DRDE-06 (accession no. EF210157.2) and a polyclonal CHIKV antibody which was raised in rabbit against the whole virus particle were gifted by Dr. M. M. Parida, DRDE, Gwalior, India. Cells were maintained in Dulbecco’s modified Eagle’s medium (DMEM; PAN Biotech, Germany) supplemented with 5% Fetal bovine serum (FBS; PAN Biotech, Germany), Gentamycin, and Penicillin-Streptomycin (Sigma, USA). C6/36 cells were maintained in MEM with 10% FBS. A polyclonal antibody raised in rabbit against 18 mer peptide of nsP2 protein was developed by us (unpublished data) and GAPDH antibody was procured from Imgenex India, Bhubaneswar, India.

### Chikungunya Virus Infection and Cellular Cytotoxicity Assay

Once the Vero cells attained 100% confluency in 35 mm cell culture dishes, cells were infected with S-27 or DRDE-06 strains of CHIKV with multiplicity of infection −1 (MOI 1) as described earlier [19]. Samples were collected at different time post infection according to the assay.

Infected cells were examined and pictures were taken at 0, 4, 8, 12, 16, 20, and 24 hours post infection (hpi) for the detection of CPE. Similarly CPE was observed at every one hour interval from 4–8 hpi for both the viruses to get more idea about the progress of infection. Cytotoxicity assay was carried out by Trypan blue exclusion method [20] where mock and virus infected cells were collected at 0, 4, 8, 12, 16, 20, and 24 hpi. After staining, viable and dead cells were counted and percentages of dead cells were considered for comparison. The standard deviations were calculated from the results of three independent experiments.

### Western Blot

Protein expression was examined by Western blot analysis according to the procedure described earlier [21]. In brief, equal numbers of Vero and C6/36 cells were infected with either S-27 or DRDE-06 with MOI 0.1, 1 and 2 and cells were harvested at different hpi according to the experiments. Cells were lysed using equal volume of lysis buffer containing 8 M urea, 2 M thiourea and 4% CHAPS [22]. Equal volume of lysate was separated on 10% SDS-polyacrylamide gel and blotted on to PVDF membrane. Protein expression was checked with nsP2 antibody (1∶3000 dilution), CHIKV antibody (1∶3000 dilution) and the same was reprobed with GAPDH antibody (1∶15,000 dilution) to use as loading control. The intensity of the nsP2 protein bands in Vero cells were quantified from three independent experiments using Quantity One software (Bio Rad, USA).

### Plaque Assay

Plaque assay was performed according to the procedure mentioned before [21]. After viral infection, the dishes were overlaid with methyl cellulose and maintained at 37°C_._ The cells were fixed at 24, 48, 72, 96, and 120 hpi and plaque diameters were measured from 10 randomly selected plaques for each time point using Leica Application Suite Advanced Fluorescence Software (LASAF) V.1.8.1. Plaque size differences were statistically analysed using the non-parametric Mann Whitney test. *P* values <0.05 were considered significant.

### Growth Kinetics

Vero cells were infected with the two virus strains with MOI 1 as mentioned above. Cell culture supernatants were collected at 0, 4, 8, 12, 16, 20, and 24 hpi and virus yield was titrated using plaque assay [21], [23]. Three independent experiments were performed and mean value was calculated for representation.

### Phylogenetic Analysis

The nucleotide sequences (full or partial) of 106 CHIKV isolates including S-27 and DRDE-06 were obtained from GenBank, NCBI and organized according to the year and place of isolation for the analysis. The phylogenetic tree was constructed using Mega 5 software (Arizona State University, USA) with the maximum-likelihood method with Tamura Nei model, nearest-neighbor-interchange and 1000 number of Bootstrap Replication [24].

### Mutational Analysis of Structural and Non-structural Proteins

The structural (n = 273) and non-structural (n = 157) protein sequences of CHIKV were also organized and aligned as mentioned above and mutations were checked. Strains were broadly classified in two groups, old strains, isolated in between 1952 to 2004 and new outbreak strains, isolated in between 2005 to 2011. Number of mutations present in a particular strain was calculated in comparison to the prototype S-27 strain. The amino acid changes which appeared during recent CHIKV outbreak and those observed mutations were checked for their presence in old and new groups of outbreak strains.

## Results

### Differences in Biological Phenotypes

**Figure 1:**
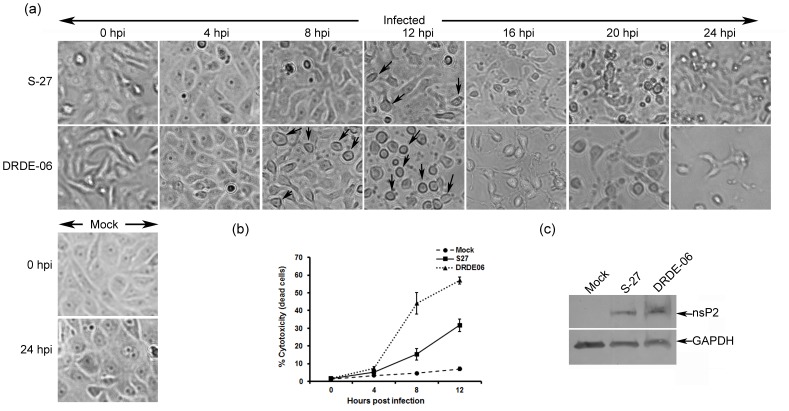
Comparison of Biological phenotypic characteristics of S-27 and DRDE-06 Chikungunya virus strains. Vero cells were infected with the viral strains with MOI 1. (a) Cytopathic effects (CPE) were observed under microscope (Magnification −20×) at different time points and arrows are indicating the cell showing CPE (at early time points only). (b) The cytotoxicity was calculated by counting the live and dead cells by trypan blue stains from three independent experiments. (c) Infected cells were harvested at 16 hours post infection (hpi) for S-27 and at 8 hpi for DRDE-06 based on the observation of highest CPE. Viral protein expression was checked in Western Blot using nsP2 antibody. GAPDH was used as a loading control.

In order to perform an *in vitro* comparison between the CHIKV prototype strain, S-27 and DRDE-06, the Vero cells were infected with the viruses (MOI 1) as mentioned above and the differences in biological phenotypes were assessed by observing CPE and calculating the cytotoxicity. As shown in Figure 1a, no CPE was observed for both the viruses up to 4 hpi. Further, almost no change was observed at 8 hpi in case of S-27 infected Vero cells, whereas changes were very prominent in DRDE-06 infection at the same time. In order to understand progression of infection better, CPE pictures were taken at every one hour interval from 4–8 hpi and observed that cell morphology actually started changing from 8 hpi for S-27 but from 5 hpi onwards for DRDE-06 (Figure S1). In addition, few rounded and detached cells were observed at 16 hpi for S-27, whereas similar characteristics were observed at 8 hpi for DRDE-06 (Figure 1a). In S-27 infected cells, maximum CPE was observed at 24 hpi, however, in DRDE-06 almost 70% cells were lysed in between 12 to 16 hpi (Figure 1a). Moreover, the comparison of cytotoxicity showed that it was nearly double in case of DRDE-06 in reference to S-27 at each time point (Figure 1b). Therefore, it might be suggested that these two viruses are biologically and phenotypically different and the 2006 Indian outbreak strain might have the ability to replicate faster than the CHIKV prototype strain. Further, to confirm viral infection, Western blot was performed which showed CHIKV nsP2 expression in S-27 and DRDE-06 infected cells whereas it was not observed in mock infected cells (Figure 1c).

### Bigger Plaques (60%) for DRDE-06

**Figure 2:**
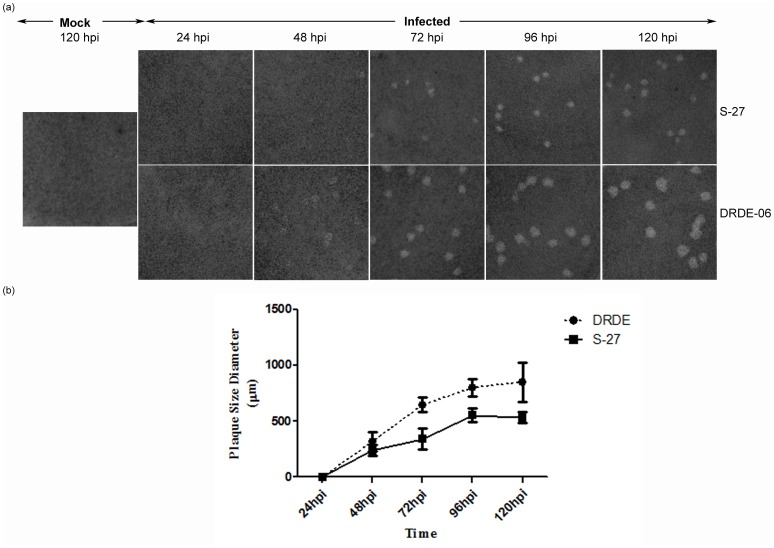
Proliferation kinetics of S-27 and DRDE-06 Chikungunya virus strains. Plaque assay was carried out in Vero cells (a) Representative pictures showings the progressive plaque formation at different time points. (b) Average plaque sizes at different time points were calculated based on the diameter of 10 randomly picked plaques of three independent experiment (p<0.05).

To confirm the observed biological and phenotypic differences between the two viruses, proliferation kinetics were carried out by plaque assay which showed visible plaques in S-27 infected cells only from 72 hpi, whereas it was visible from 48 hpi in case of DRDE-06 (Figure 2a). After comparing the plaques developed at different time points, it was noticed that both the strains of viruses displayed plaques of clear morphology but the mean plaque diameters were around 60% bigger in case of the DRDE-06 (p = 0.0001; Figure 2b). This data further underline the fact that DRDE-06 might have higher infectivity than the prototype strain S-27.

### Early Release of Progeny Viruses for DRDE-06

**Figure 3:**
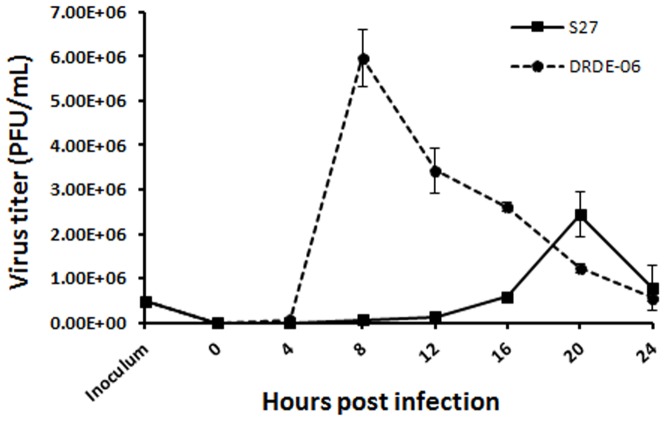
Growth kinetics of S-27 and DRDE-06 Chikungunya virus strains. Vero cells were infected with viruses and supernatants were collected at different time intervals. Growth curves were obtained by plotting viral titres present at each time. Virus titres were calculated from three independent experiments.

To elucidate the differences in fundamental biological features of these two viruses, growth kinetic assay was performed. As shown in Figure 3, it was observed that S-27 had highest release of virus particles at 20 hpi (2.43E+06 PFU/ml), on the contrary, DRDE-06 displayed peak release of viruses at 8 hpi with more than double (6.00E+06 PFU/ml) progeny viruses in comparison to S-27. As the supernatants were collected at every 4 hr intervals, thus the titer of the viruses at a particular hpi means the total virus particle released within 4 hrs. Although, virus infections were performed with same MOI of these two viruses, but the viral growth kinetics were remarkably different. In consistent with the earlier results, the growth kinetics also demonstrated that DRDE-06 produced new virus particles at a remarkable pace in between the first 4–8 hpi and rapidly reached to the highest titre (6.00E+06 PFU/ml).

### Comparison of Viral Protein Expression Level

**Figure 4:**
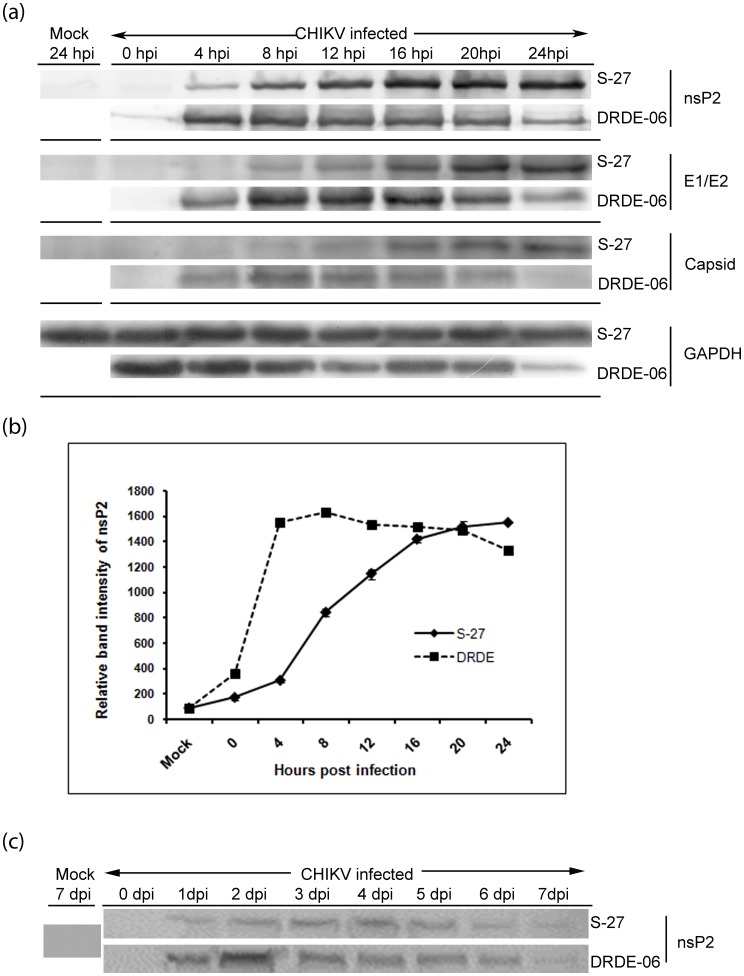
Expression pattern of Chikungunya viral proteins. Vero cells were infected with S-27 and DRDE-06 with MOI 1 and cells were harvested at different times post infection (hpi - hours post infection). (a) Viral proteins (nsP2, E1/E2 and capsid) detected by Western blot by using respective antibodies. GAPDH was used as a loading control. (b) Representation of nsP2 protein expression quantified by Quantity One software. Values were calculated from three independent experiments. (c) C6/36 cells were infected with S-27 and DRDE-06 with MOI 1 and cells were harvested at different days post infection (dpi). Viral protein nsP2 was detected by Western blot using nsP2 polyclonal antibody.

As the outbreak strain displayed all biological and phenotypic characteristics much faster than the prototype strain, we studied nsP2 and structural (E1/E2 and capsid) protein expressions by Western blot (Figure 4a) and quantitated nsP2 protein expression levels by Western blot (Figure 4b) as well as Flow cytometric analysis (data not shown). As demonstrated in Figure 4a and Figure 4b, the overall nsP2 protein levels were lower in S-27 infected cells and it reached to the peak slowly at around 20–24 hpi, whereas, much higher level of nsP2 protein expression was detected from early time points in DRDE-06 and it reached to the peak rapidly at around 8 hpi. The structural protein expression also followed the similar pattern (Figure 4a). Next, we performed similar experiments with multiple MOIs (0.1, 1 and 2) from 0 to 24 hpi at every four hour intervals to get a better glimpse of the dynamic properties of these two viruses in terms of nsP2 viral protein expression. For all the MOIs, expression of nsP2 was earlier in DRDE-06 as compared to S-27 (data not shown) and differences were very prominent in early phase of infection (up to 8 hpi). In order to understand progression of infection better in early phase, cells were collected at every one hour intervals from 0–7 hpi and Western blot was performed similarly. It was observed that nsP2 protein was detected at very low level from 1 hpi onwards and displayed very slow gradual increase over time in case of S-27 (Figure S2, Figure 4a and Figure 4b). On the contrary, in case of DRDE-06, a prominent band of nsP2 was observed at even 0 hpi (immediately after 1.5 hr adsorption) and from 2 hpi onwards the level of expression of nsP2 increased drastically (Figure S2, Figure 4a and Figure 4b). Since, all the analyses in mammalian cells showed early and higher infectivity of DRDE-06 in a small span of time as compared to S-27, it was important to know whether the outbreak strain can exhibit the similar pattern of infection in mosquito cells. Interestingly, the highest level of nsP2 expression was observed on day four after viral infection in C6/36 mosquito cells for S-27, while the same was observed on day two in case of DRDE-06 (Figure 4c). Over all, these results clearly confirmed that the prototype strain and the 2006 Indian outbreak strain both exhibited remarkably different pattern of infection in mammalian as well as in mosquito cells.

### Phylogenetic Analysis

All the experimental observations established the fact that there were differences in infection pattern between these two CHIKV strains. This prompted us to analyse the nucleotide and protein sequences of these two strains along with several other global isolates to find out the genetic changes which might be responsible for observed phenotypic differences.

**Figure 5:**
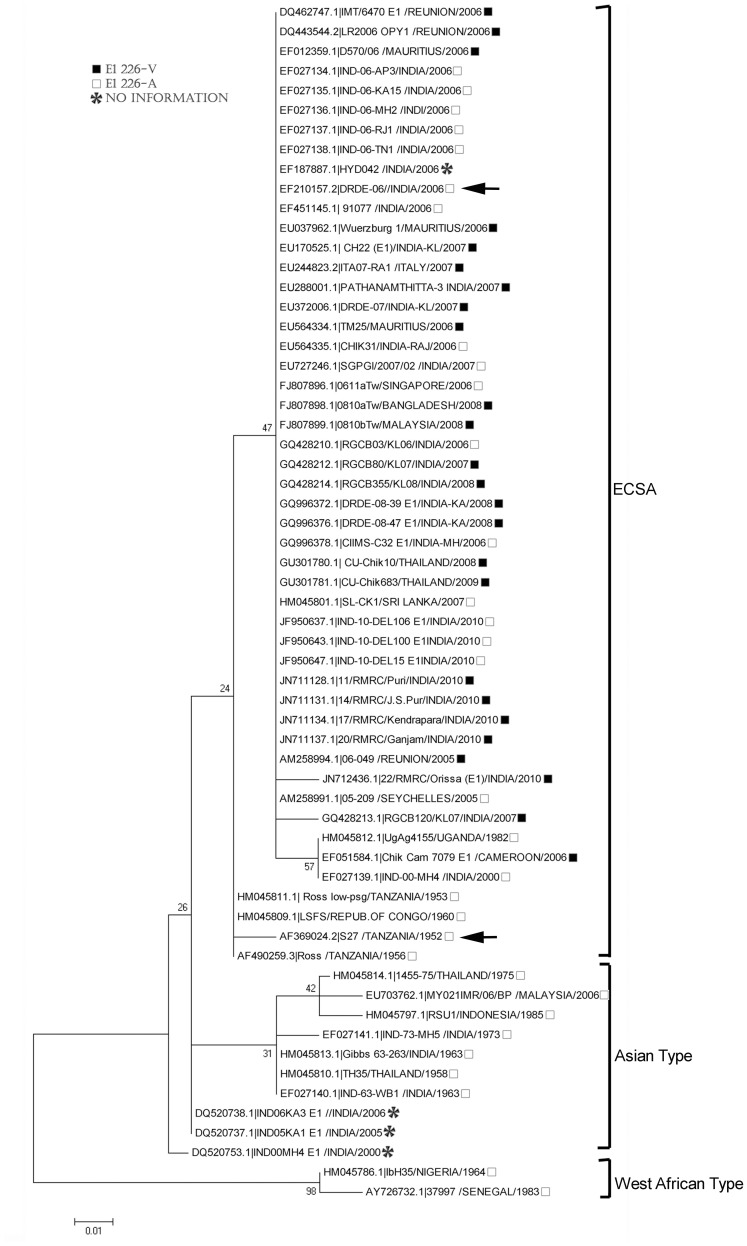
Phylogenetic analysis of E1 sequences of 61 representative Chikungunya viruses global isolates from the year 1952 to 2011. The unrooted tree was constructed using Neighbor-Joining method with 1000 bootstrap value. Numbers along with branches indicate bootstrap values. Scale bar indicates nucleotides substitutions per site. The presence/absence of E1-A226V mutation is depicted by black filled square and open square. *indicates the unavailability of sequence information for E1-A226V mutation and Arrow indicates strains used in this study. (The full Phylogenetic tree of 106 global isolates from the year 1952 to 2011 may be viewed in Figure S3).

The E1 nucleotide sequences (full or partial) of 106 strains (Table S1) were aligned and phylogenetic tree was constructed to determine the progenitor phylogroup of all the outbreak strains circulated worldwide from 1952 to 2011. Our analysis clearly demonstrated that all recent outbreak strains including DRDE-06 clustered in a homogeneous clade within the major group of ECSA type of CHIKV, however, prototype strain, S-27 was clustered with the old ECSA type isolates (Figure 5 and Figure S3). Since, previous reports suggested the appearance of E1-A226V mutation at the end of year 2005 in Reunion Island and also in different other locations at different times from 2006 onwards, we were interested to look for the presence of this change in the E1 protein in all our studied populations. The analyses showed that E1-A226V mutation was not present in all the strains isolated during 2005 to 2011 (Table 1 and Table S4). Altogether, ∼53% of the recent outbreak strains (2005 to 2011) showed the presence of E1-A226V mutation, however other ∼46% including DRDE-06 did not have this mutation (Table 1 and Table S4).

**Table 1:** Year wise distribution of E1-A226V mutant genotype of Chikungunya viruses isolated from the year 1952 to 2011.

Year	E1-226A	E1-226V	E1-226G	No SequenceInformation	Total CHIKV Isolates
**1952 to 2004**	36	0		1	37
**2005**	3	0			3
**2006**	40	7		4	51
**2007**	11	15	1	7	34
**2008**	11	54			65
**2009**	16	7		5	28
**2010**	20	26		1	47
**2011**	0	8			8
**All**	137	117	1	18	273
**2005 to 2011**	101	117	1	0	219
**%**	46.119	53.42	0.46		100

### Consistent Presence of Fifteen Mutations in Recent Outbreak Strains

The mutational analysis of structural and non-structural protein sequences of prototype strain S-27 and DRDE-06 was carried out and it was observed that there were total nineteen mutations in DRDE-06. Eight mutations (two in C, P23S, V27I; three in E2, T637M, S700T, V711A; one in 6K, V756I, and two in E1, D1093E, V1167A) were identified in the structural proteins (Table S2) and eleven mutations (four in nsP1, T128K, M314L, T376M and Q488R; two in nsP2, S589N and A1328V; three in nsP3, Y1550H, T1670I, P1804S, and two in nsP4, T1938A, T2117A) were detected in non-structural proteins (Table S3). Furthermore, in-depth mutational analysis of structural (n = 273) and non-structural protein sequences (n = 157) of global isolates with reference to prototype strain S-27 revealed that CHIKV strains were acquiring more number of mutations till 2001, whereas very few mutations were acquired after 2001 or 2005 and these mutations were maintained in almost all recent outbreak isolates (2005 to 2011) (Table S2 and Table S3). Few mutations were sporadic and location specific, but majority of those new mutations were present up to the year 2011. Among the nineteen mutations mentioned above for DRDE-06, except four (one in E2, S700T; one in E1, V1167A; two in nsP1, M314L and Q488R) all other fifteen mutations were consistently present in almost all recent outbreak strains considered in our mutational analysis (Table S2 and Table S3).

## Discussion

Recent massive outbreaks (2005 to 2011) of CHIKV in different parts of the world emphasizes the need of further investigation to understand the factors contributing to the severity of the disease and its rapid spread [2]–[5], [10], [13], [19], [25].

In this study, we analyse the biological phenotypic differences of the CHIKV prototype strain, S-27 and DRDE-06, one of the strains isolated from Southern India during recent outbreak in 2006 [19], [25]. This investigation demonstrate that the CHIKV outbreak strain has ability to replicate much faster and produce more than double number of progeny viruses with reference to prototype strain in mammalian cell culture experiments. Further, the observation of CPE, detection of non-structural and structural proteins and viral proliferation assay support the enhanced and faster infection rate of the outbreak strain, DRDE-06. Similarly, early expression of nsP2 is observed for DRDE-06 CHIKV strain in mosquito cell line, C6/36, indicating its higher and faster infectivity in vector as well. Moreover, our mutational analysis of whole genome of 273 global CHIKV isolates provides possible association of the observed phenotypic changes with genetic mutations.

It was reported in H5N1, Avian Influenza virus that the higher rate of replication of the HK483 strain was directly related to rapid evasion of host immune system and the extreme pathogenicity in the host [26]. Similar observations were also noticed for Adenovirus and Coxsackie virus which demonstrated that higher rate of replication is often linked to higher pathogenicity or virulency during infection [23], [27]. Therefore, it may be speculated that the faster rate of replication and infection of the recent CHIKV outbreak strain, DRDE-06 may be correlated with its high pathogenicity or virulency through rapid evasion of host immune responses. However, the genetic changes responsible for the phenotypic differences are not properly understood. Our study indicates that nineteen mutations present in DRDE-06 may be associated with the biological and phenotypic differences which are observed in this study. There are few residues which are predicted to have phosphorylation potential in non-structural proteins (T128K, T376M, T1670I, T2117A) and few others are predicted to be involved in binding to important domains of different host proteins in both structural (V27I, E637N, V756I, D1093E) and non-structural proteins (S589N, A1328Y, Y1550H, T1670I, T1938A). This analysis indicates that these mutant residues may have role in changing the expression profile or stability of the viral proteins or their binding partners during infection which need further investigation.

Earlier reports demonstrated that E1-A226V mutant CHIKV strain shows better fitness in a new vector *Aedes albopictus*, rather than its typical vector, *Aedes aegypti.* This report also indicates the possibility of its adaptation to *Aedes albopictus* that may explain the cause of recent epidemics by increasing the spread of this virus [28], [29]. Interestingly, DRDE-06, one of the outbreak strain used in this study does not have E1-A226V mutation. In addition, our mutational analysis shows that almost ∼53% of the total recent outbreak isolates from 2005 to 2011 (n = 219) have the E1-A226V mutation, but rest of the strains (∼46%) lack this mutation, indicating that the strains with and without E1-A226V mutation were co-circulating during recent extensive CHIKV outbreaks in different countries such as Reunion Island, India, Bangladesh, Thailand, Malaysia, Japan, Italy, Australia, Cambodia, Mauritius, Singapore, SriLanka, China and France [5], [10]–[12], [14], [17], [18], [25], [30]–[33]. Moreover, our mutational analyses of 273 global CHIKV isolates reveal that 15 mutations are consistently present in all outbreak strains including DRDE-06 compared to the old strains isolated before year 2004. Therefore, it may appear that CHIKV outbreak strains without E1-A226V mutation might have already evolved as highly infective strains of epidemic potential through other genetic changes, however, the acquisition of E1-A226V mutation, probably helped the virus to cross host species barrier and increased the transmissibility worldwide within a short span of time. Further studies with different mutant viruses will help to identify the unique determinant for the association of genotypic changes with observed different patterns of infection and it may further address the questions related to virus virulency and disease pathogenesis, if any, by using animal model.

In conclusion, chikungunya outbreak strain, DRDE-06 exhibits different pattern of infection as compared to prototype strain, S-27. This might be associated to some specific mutations, as observed in genome wide mutational analysis in DRDE-06, which emphasizes the need of further experimental investigations.

## Supporting Information

Figure S1 **Comparison of Biological phenotypic characteristics of S-27 and DRDE-06 Chikungunya virus strains.** Vero cells were infected with the viral strains with MOI 1. Cytopathic effects (CPE) were observed under microscope (Magnification −20X) at every one hour interval from 5–8 hpi for both the viruses.
(TIF)

Figure S2 **Expression pattern of Chikungunya viral protein.** Vero cells were infected with S-27 and DRDE-06 with MOI 2 and cells were harvested at every one hour interval from 0–7 hpi (hpi - hours post infection). Expression pattern of CHIKV nsp2 protein was checked by Western blot. GAPDH was used as a loading control.
(TIF)

Figure S3 **Phylogenetic analysis of E1 sequences of 106 Chikungunya viruses global isolates from the year 1952 to 2011.** The unrooted tree was constructed using Neighbor-Joining method with 1000 bootstrap value. Numbers along with branches indicate bootstrap values. Scale bar indicates nucleotides substitutions per site.
(PDF)

Table S1 **Details of the CHIKV genome sequences of different global strains along with accession numbers (n = 106) used in this study.**
(PDF)

Table S2 **Details of the CHIKV Structural protein sequences of different global strains along with accession numbers (n = 273) used in this study.** Amino acid position of consistent mutations present in this region is shown along with alignment.
(PDF)

Table S3 **Details of the CHIKV Non-Structural protein sequences of different global strains along with accession numbers (n = 157) used in this study.** Amino acid position of consistent mutations present in this region is shown along with alignment.
(PDF)

Table S4 **Showing the alignment of E1 structural protein sequence from amino acid position 217 (1026 aa for polyprotein) to 236 (1045 aa for polyprotein) of different global strains used in this study.** The position of E1-A226V (1035aa for polyprotein) mutation has been highlighted to show the occurrence of this mutation.
(PDF)
