# Supplementary figures and images for: A Novel 2006 Indian Outbreak Strain of Chikungunya Virus Exhibits Different Pattern of Infection as Compared to Prototype Strain
Source: PLoS One. 2014 Jan 20;9(1):e85714. doi: 10.1371/journal.pone.0085714 (PMC3896419; doi:10.1371/journal.pone.0085714)

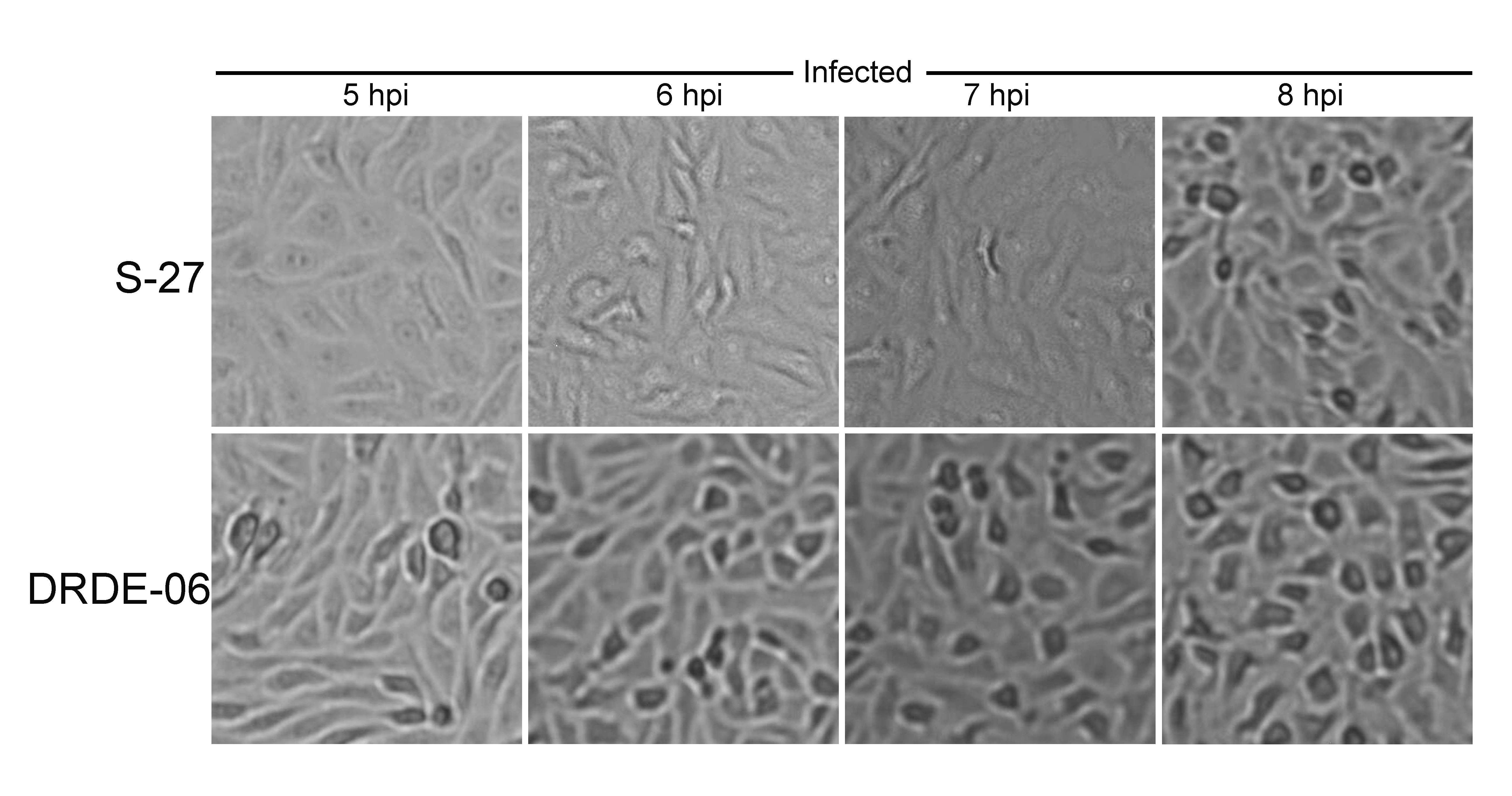

Supplement: Figure S1 — Comparison of Biological phenotypic characteristics of S-27 and DRDE-06 Chikungunya virus strains. Vero cells were infected with the viral strains with MOI 1. Cytopathic effects (CPE) were observed under microscope (Magnification −20X) at every one hour interval from 5–8 hpi for both the viruses. (TIF) [file pone.0085714.s001.tif]

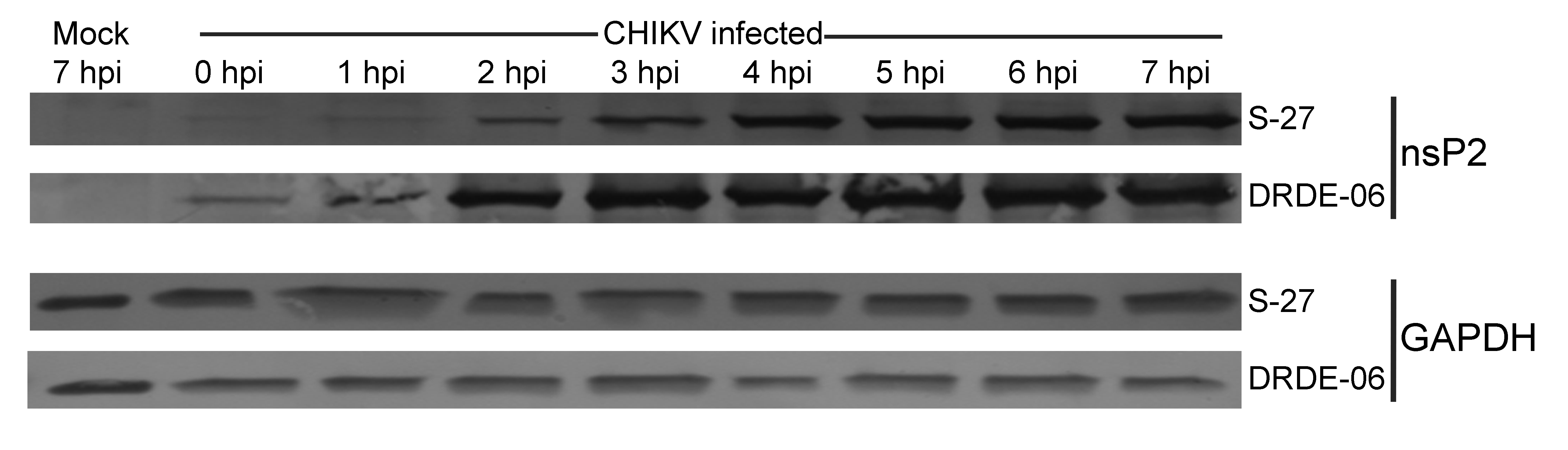

Supplement: Figure S2 — Expression pattern of Chikungunya viral protein. Vero cells were infected with S-27 and DRDE-06 with MOI 2 and cells were harvested at every one hour interval from 0–7 hpi (hpi - hours post infection). Expression pattern of CHIKV nsp2 protein was checked by Western blot. GAPDH was used as a loading control. (TIF) [file pone.0085714.s002.tif]
