# Supplementary material for: A Novel 2006 Indian Outbreak Strain of Chikungunya Virus Exhibits Different Pattern of Infection as Compared to Prototype Strain
Source: PLoS One. 2014 Jan 20;9(1):e85714. doi: 10.1371/journal.pone.0085714 (PMC3896419; doi:10.1371/journal.pone.0085714)

Figure S1: Phylogenetic tree of Chikungunya viruses generated by Neighbor-Joining method based on E1 sequences of 106 global isolates from the year 1952 to 2011.

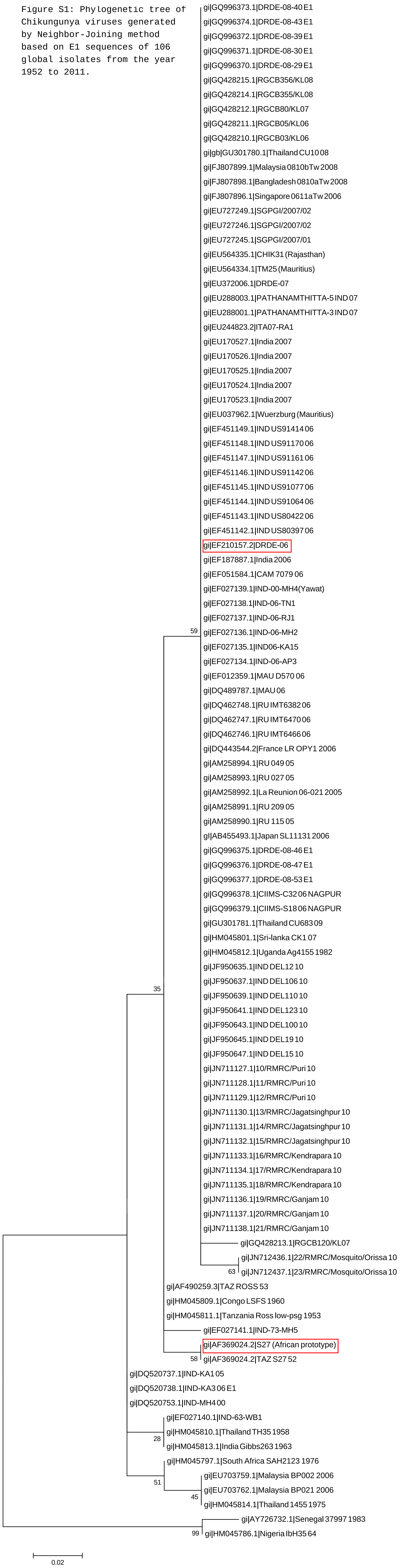

Supplement: Figure S3 — Phylogenetic analysis of E1 sequences of 106 Chikungunya viruses global isolates from the year 1952 to 2011. The unrooted tree was constructed using Neighbor-Joining method with 1000 bootstrap value. Numbers along with branches indicate bootstrap values. Scale bar indicates nucleotides substitutions per site. (PDF) [file pone.0085714.s003.pdf]
