# Supplementary material for: A Novel 2006 Indian Outbreak Strain of Chikungunya Virus Exhibits Different Pattern of Infection as Compared to Prototype Strain
Source: PLoS One. 2014 Jan 20;9(1):e85714. doi: 10.1371/journal.pone.0085714 (PMC3896419; doi:10.1371/journal.pone.0085714)
Supplement: Table S1 — Details of the CHIKV genome sequences of different global strains along with accession numbers (n = 106) used in this study. (PDF) [file pone.0085714.s004.pdf]

Table S1: Details of the CHIKV genome sequences of different strains along with accession numbers (n=106) used in this study

Sr. No	Accession No	Year	Country /strain name
1	AF369024.2	1952	S27_(African_prototype)
2	AF369024.2	1952	TAZ_S27_52
3	HM045811.1	1953	Tanzania_Ross_low-psg_1953
4	AF490259.3	1953	TAZ_ROSS_53
5	HM045810.1	1958	Thailand_TH35_1958
6	HM045809.1	1960	Congo_LSFS_1960
7	EF027140.1	1963	IND-63-WB1
8	HM045813.1	1963	India_Gibbs263_1963
9	HM045786.1	1964	Nigeria_IbH35_64
10	EF027141.1	1973	IND-73-MH5
11	HM045814.1	1975	Thailand_1455_1975
12	HM045797.1	1976	South_Africa_SAH2123_1976
13	HM045812.1	1982	Uganda_Ag4155_1982
14	AY726732.1	1983	Senegal_37997_1983
15	DQ520753.1	2000	IND-MH4_00
16	EF027139.1	2004	IND-00-MH4(Yawat)
17	DQ520737.1	2005	IND-KA1_05
18	AM258992.1	2005	La_Reunion_06-021_2005
19	AM258993.1	2005	RU_027_05
20	AM258994.1	2005	RU_049_05
21	AM258990.1	2005	RU_115_05
22	AM258991.1	2005	RU_209_05
23	EF051584.1	2006	CAM_7079_06
24	EU564335.1	2006	CHIK31_(Rajasthan)
25	GQ996378.1	2006	CIIMS-C32_06_NAGPUR
26	GQ996379.1	2006	CIIMS-S18_06_NAGPUR
27	EF210157.2	2006	DRDE-06
28	DQ443544.2	2006	France_LR_OPY1_2006
29	EF451142.1	2006	IND_US80397_06
30	EF451143.1	2006	IND_US80422_06
31	EF451144.1	2006	IND_US91064_06
32	EF451145.1	2006	IND_US91077_06
33	EF451146.1	2006	IND_US91142_06
34	EF451147.1	2006	IND_US91161_06
35	EF451148.1	2006	IND_US91170_06
36	EF451149.1	2006	IND_US91414_06
37	EF027134.1	2006	IND-06-AP3
38	EF027135.1	2006	IND06-KA15
39	EF027136.1	2006	IND-06-MH2
40	EF027137.1	2006	IND-06-RJ1
41	EF027138.1	2006	IND-06-TN1
42	EF187887.1	2006	India_2006
43	DQ520738.1	2006	IND-KA3_06_E1
44	AB455493.1	2006	Japan_SL11131_2006
45	EU703759.1	2006	Malaysia_BP002_2006
46	EU703762.1	2006	Malaysia_BP021_2006
47	DQ489787.1	2006	MAU_06
48	EF012359.1	2006	MAU_D570_06

49	GQ428210.1	2006	RGCB03/KL06
50	GQ428211.1	2006	RGCB05/KL06
51	DQ462748.1	2006	RU_IMT6382_06
52	DQ462746.1	2006	RU_IMT6466_06
53	DQ462747.1	2006	RU_IMT6470_06
54	FJ807896.1	2006	Singapore_0611aTw_2006
55	EU564334.1	2006	TM25_(Mauritius)
56	EU372006.1	2007	DRDE-07
57	EU170523.1	2007	India_2007
58	EU170524.1	2007	India_2007
59	EU170525.1	2007	India_2007
60	EU170526.1	2007	India_2007
61	EU170527.1	2007	India_2007
62	EU244823.2	2007	ITA07-RA1
63	EU288001.1	2007	PATHANAMTHITTA-3_IND_07
64	EU288003.1	2007	PATHANAMTHITTA-5_IND_07
65	GQ428213.1	2007	RGCB120/KL07
66	GQ428212.1	2007	RGCB80/KL07
67	EU727245.1	2007	SGPGI/2007/01
68	EU727246.1	2007	SGPGI/2007/02
69	EU727249.1	2007	SGPGI/2007/02
70	HM045801.1	2007	Sri-lanka_CK1_07
71	EU037962.1	2007	Wuerzburg_(Mauritius)
72	FJ807898.1	2008	Bangladesh_0810aTw_2008
73	GQ996370.1	2008	DRDE-08-29_E1
74	GQ996371.1	2008	DRDE-08-30_E1
75	GQ996372.1	2008	DRDE-08-39_E1
76	GQ996373.1	2008	DRDE-08-40_E1
77	GQ996374.1	2008	DRDE-08-43_E1
78	GQ996375.1	2008	DRDE-08-46_E1
79	GQ996376.1	2008	DRDE-08-47_E1
80	GQ996377.1	2008	DRDE-08-53_E1
81	FJ807899.1	2008	Malaysia_0810bTw_2008
82	GQ428214.1	2008	RGCB355/KL08
83	GQ428215.1	2008	RGCB356/KL08
84	GU301780.1	2008	Thailand_CU10_08
85	GU301781.1	2009	Thailand_CU683_09
86	JN711127.1	2010	10/RMRC/Puri_10
87	JN711128.1	2010	11/RMRC/Puri_10
88	JN711129.1	2010	12/RMRC/Puri_10
89	JN711130.1	2010	13/RMRC/Jagatsinghpur_10
90	JN711131.1	2010	14/RMRC/Jagatsinghpur_10
91	JN711132.1	2010	15/RMRC/Jagatsinghpur_10
92	JN711133.1	2010	16/RMRC/Kendrapara_10
93	JN711134.1	2010	17/RMRC/Kendrapara_10
94	JN711135.1	2010	18/RMRC/Kendrapara_10
95	JN711136.1	2010	19/RMRC/Ganjam_10
96	JN711137.1	2010	20/RMRC/Ganjam_10
97	JN711138.1	2010	21/RMRC/Ganjam_10
98	JN712436.1	2010	22/RMRC/Mosquito/Orissa_10
99	JN712437.1	2010	23/RMRC/Mosquito/Orissa_10
100	JF950643.1	2010	IND_DEL100_10

101	JF950637.1	2010	IND_DEL106_10
102	JF950639.1	2010	IND_DEL110_10
103	JF950635.1	2010	IND_DEL12_10
104	JF950641.1	2010	IND_DEL123_10
105	JF950647.1	2010	IND_DEL15_10
106	JF950645.1	2010	IND_DEL19_10
