# Supplementary material for: A Novel 2006 Indian Outbreak Strain of Chikungunya Virus Exhibits Different Pattern of Infection as Compared to Prototype Strain
Source: PLoS One. 2014 Jan 20;9(1):e85714. doi: 10.1371/journal.pone.0085714 (PMC3896419; doi:10.1371/journal.pone.0085714)
Supplement: Table S2 — Details of the CHIKV Structural protein sequences of different global strains along with accession numbers (n = 273) used in this study. Amino acid position of consistent mutations present in this region is shown along with alignment. (PDF) [file pone.0085714.s005.pdf]

Table S2. Details of the CHIKV Structural protein sequences of different global strains along with accession numbers (n=273) used in this study. Amino acid position of consistent mutations present in this region is shown along with alignment.

	**Amino acid position of structral polyprotein**						
							1
				6	7	7	0
		2	2	3	1	5	9
**Sr. No**	**Strain Details**	3	7	7	1	6	3
**1**	**1953 South Africa gi|AAN05102.2|S27 (African prototype)**	**P**	**V**	**T**	**V**	**V**	**D**
2	1953 TANZANIA gi|ADG95932.1|TANZANIA-1953-Ross LOW-psg-structural polyprotein Chikungunya virus	.	.	.	.	.	.
3	1956 SOUTH AFRICA gi|ADG95897.1|SOUTH AFRICA-1956-Vereenining-structural polyprotein Chikungunya virus	.	.	.	.	.	.
4	1958 THAILAND gi|ADG95930.1|THAILAND-1958-TH35-structural polyprotein Chikungunya virus	.	.	.	.	.	.
5	1960 CONGO gi|ADG95928.1|CONGO-1960-LSFS-structural polyprotein Chikungunya virus	.	.	.	.	.	.
6	1962 ANGOLA gi|ADG95956.1|ANGOLA-1962-Angola M2022-structural polyprotein Chikungunya virus	.	.	.	.	.	.
7	1963 INDIA gi|ADG95917.1|IND-1963-I-634029-structural polyprotein Chikungunya virus	.	.	.	.	.	.
8	1963 INDIA gi|ADG95936.1|IND-1963-Gibbs 63-263-structural polyprotein Chikungunya virus	.	.	.	.	.	.
9	1963 SENEGAL gi|ADG95952.1|SENEGAL-1963-A301-structural polyprotein Chikungunya virus	.	.	.	.	.	.
10	1964 NIGERIA gi|ADG95885.1|NIGERIA-1964-IbH35-structural polyprotein Chikungunya virus	.	.	.	.	A	.
11	1965 NIGERIA gi|ADG95924.1|NIGERIA-1965-IbAn4824-structural polyprotein Chikungunya virus	.	.	.	.	A	.
12	1966 SENEGAL gi|ADG95883.1|SENEGAL-1966-PM2951-structural polyprotein Chikungunya virus	.	.	.	.	A	.
13	1966 SENEGAL gi|ADG95907.1|SENEGAL-1966-SH2830-structural polyprotein Chikungunya virus	.	.	.	.	A	.
14	1966 SENEGAL gi|ADG95942.1|SENEGAL-1966-SH 3013-structural polyprotein Chikungunya virus	.	.	.	.	A	.
15	1973 INDIA gi|ADG95889.1|IND-1973-PO731460-structural polyprotein Chikungunya virus	.	.	.	.	A	.
16	1975 THIALAND gi|ADG95938.1|THIALAND-1975-1455-75-structural polyprotein Chikungunya virus	.	.	.	.	.	.
17	1976 SOUTH AFRICA gi|ADG95902.1|SOUTH AFRICA-1976-SAH2123-structural polyprotein Chikungunya virus	.	.	.	.	.	.
18	1976 SOUTH AFRICA gi|ADG95921.1|SOUTH AFRICA-1976-AR 18211-structural polyprotein Chikungunya virus	.	.	.	.	.	.
19	1978 CENTRAL AFRICAN REPUBLIC gi|ADG95954.1|CENTRAL AFRICAN REPUBLIC-1978-HB78-structural polyprotein Chikungunya	.	.	.	.	.	.
20	1978 THIALAND gi|ADG95926.1|THIALAND-1978-structural polyprotein Chikungunya virus	.	.	.	.	.	.
21	1979 SENEGAL gi|ADG95940.1|SENEGAL-1979-ArD 30237-structural polyprotein Chikungunya virus	.	.	.	.	A	.
22	1981 COTE DIVOIRE gi|ADG95946.1|COTE DIVOIRE-1981-ArA 2657-structural polyprotein Chikungunya virus	.	.	.	.	A	.
23	1982 UGNADA gi|ADG95934.1|UGNADA-1982-UgAG4155-structural polyprotein Chikungunya virus	.	.	.	.	.	.
24	1983 INDONESIA gi|ADG95895.1|INDONESIA-1983-JKT23574-structural polyprotein Chikungunya virus	.	.	.	.	.	.
25	1984 CENTRAL AFRICAN REPUBLIC gi|ADG95881.1|CENTRAL AFRICA REPUBLIC-1984-DakAR B 16878-structural polyprotein Chik	.	.	.	.	.	.
26	1985 INDONESIA gi|ADG95906.1|INDONESIA-1985-RSU1-structural polyprotein Chikungunya virus	.	.	.	.	.	.
27	1985 PHILIPPINNES gi|ADG95893.1|PHILIPPINNES-1985-PhH15483-structural polyprotein Chikungunya virus	.	.	.	.	.	.
28	1985 PHILIPPINNES gi|ADG95911.1|PHILIPPINES-1985-Hu/85/NR/001-structural polyprotein Chikungunya virus	.	.	.	.	.	.
29	1986 IND gi|ADG95923.1|IND-1986-ALSA-Aedes Albopictus cell line-structural polyprotein Chikungunya virus	.	.	.	.	.	.
30	1988 THIALAND gi|ADG95891.1|THAILAND-1988-6441-88-structural polyprotein Chikungunya virus	.	.	.	.	.	.
31	1993 COT DIVOIRE gi|ADG95950.1|COT DIVOIRE-1993-ArA 30548-structural polyprotein Chikungunya virus	.	.	.	.	A	.
32	1993 SENEGAL gi|ADG95948.1|SENEGAL-1993-AeD 93229-structural polyprotein Chikungunya virus	.	.	.	.	A	.
33	1995 THIALAND gi|ADG95887.1|THAILAND-1995-SV0444-95-structural polyprotein Chikungunya virus	.	.	.	.	.	.
34	1995 THIALAND gi|ADG95904.1|THAILAND-1995-CO392-95-structural polyprotein Chikungunya virus	.	.	.	.	.	.
35	1995 THIALAND gi|ADG95915.1|THAILAND-1995-K0146-95-structural polyprotein Chikungunya virus	.	.	.	.	.	.
36	2000 IND-MH gi|ABN04198.1|IND-00-MH4 (Yawat)	.	.	.	.	.	.
37	2001 JAPAN gi|YP 006491243.1|Japan-2001-S27 passeg in Aedes albopticus C6/36 cell line truncated polyprotein Chikungunya virus	.	.	.	.	.	-
38	2005 RAW UNION gi|CAJ90470.1|RU 115 05	.	.	M	A	I	E
39	2005 SENEGAL gi|ADG95944.1|SENEGAL-2005-HD 180760-structural polyprotein Chikungunya virus	.	.	.	.	A	.
40	2005 Seychelles gi|106880538|emb|CAJ90473.1| structural polyprotein Chikungunya virus	.	.	M	A	I	E
41	2006 CAMROON gi|ABN04115.1|CAM 7079 06	-	-	-	-	-	.
42	2006 IND-AP gi|ABN04188.1|IND-06-AP3	S	.	M	A	I	E
43	2006 IND-GUJ gi|ACM09907.1|IND-GUJ-2006-IND-GJ52-structural polyprotein Chikungunya virus	S	I	M	A	I	E
44	2006 IND-GUJ gi|ACM09911.1|IND-GUJ-2006-IND-GJ51-structural polyprotein Chikungunya virus	S	I	M	A	I	E
45	2006 IND-GUJ gi|ACM09913.1|IND-GUJ-2006-IND-GJ53-structural polyprotein Chikungunya virus	S	I	M	A	I	E
46	2006 IND-GUJ gi|AEA10291.1|IND-GUJ-2006-structural polyprotein Chikungunya virus	S	I	M	A	I	E
47	2006 INDIA gi|ABO40448.1|IND US91064 06	-	-	-	A	I	E
48	2006 INDIA gi|ABO40451.1|IND US91161 06	-	-	-	A	.	E
49	2006 INDIA gi|ABO40453.1|IND US91414 06	-	-	-	A	I	E
**50**	**2006 INDIA gi|ABP88822.1|DRDE-06**	**S**	**I**	**M**	**A**	**I**	**E**
51	2006 INDIA gi|ABO40446.1|IND US80397 06	-	-	-	A	I	E
52	2006 INDIA gi|ABO40447.1|IND US80422 06	-	-	-	A	I	E
53	2006 INDIA gi|ABO40449.1|IND US91077 06	-	-	-	A	I	E
54	2006 INDIA gi|ABO40450.1|IND US91142 06	-	-	-	A	I	E
55	2006 INDIA gi|ABO40452.1|IND US91170 06	-	-	-	A	I	E
56	2006 INDIA gi|AEE60791.1|IND-2006-structural polyprotein Chikungunya virus	S	I	M	A	I	E
57	2006 INDIA gi|AEE60794.1|IND-2006-structural polyprotein Chikungunya virus	S	I	M	A	I	E
58	2006 INDIA gi|AEE60795.1|IND-2006-structural polyprotein Chikungunya virus	S	I	M	A	I	E
59	2006 INDIA gi|AEE60796.1|IND-2006-structural polyprotein Chikungunya virus	S	I	M	A	I	E
60	2006 INDIA gi|AEQ59648.1|IND-2006-envelope glycoprotein 1 Chikungunya virus	-	-	-	-	-	-
61	2006 INDIA gi|AEQ59649.1|IND-2006-envelope glycoprotein 1 Chikungunya virus	-	-	-	-	-	-

	Amino acid position of structral polyprotein						1
				6	7	7	0
		2	2	3	1	5	9
**Sr. No**	**Strain Details**	3	7	7	1	6	3
**1**	**1953 South Africa gi|AAN05102.2|S27 (African prototype)**	**P**	**V**	**T**	**V**	**V**	**D**
62	2006 INDIA gi|AEQ59650.1|IND-2006-envelope glycoprotein 1 Chikungunya virus	-	-	-	-	-	-
63	2006 INDIA gi|AEQ59651.1|IND-2006-envelope glycoprotein 1 Chikungunya virus	-	-	-	-	-	-
64	2006 IND-KA gi|ABN04190.1|IND-06-KA15	S	I	M	A	I	E
65	2006 IND-KA gi|ACM09909.1|IND-KA-2006-IND-KA52-structural polyprotein Chikungunya virus	S	I	M	A	I	E
66	2006 IND-KA gi|ACM09919.1|IND-KA-2006-IND-KA51-structural polyprotein Chikungunya virus	S	I	M	A	I	E
67	2006 IND-KL gi|ACY25938.1|IND-2006-RGCB03/KL06-structural polyprotein Chikungunya virus	S	I	M	A	I	E
68	2006 IND-KL gi|ACY25940.1|IND-2006-RGCB05/KL06-structural polyprotein Chikungunya virus	S	I	M	A	I	E
69	2006 IND-KR gi|ACM09915.1|IND-KR-2006-IND-KR51-structural polyprotein Chikungunya virus	S	I	M	A	I	E
70	2006 IND-MAH gi|ACM09917.1|IND-MAH-2006-IND-MH51-structural polyprotein Chikungunya virus	S	.	M	A	I	E
71	2006 IND-MH gi|ABN04192.1|IND-06-MH2	S	I	M	A	I	E
72	2006 IND-NAGPUR gi|ADN85594.1|CIIMS-C32 2006 NAGPUR	-	-	-	-	-	E
73	2006 IND-NAGPUR gi|ADN85594.1|IND-NAGPUR-2006(CIIMS-C)-E1 protein Chikungunya virus	-	-	-	-	-	E
74	2006 IND-NAGPUR gi|ADN85595.1|CIIMS-S18 2006 NAGPUR	-	-	-	-	-	E
75	2006 IND-NAGPUR gi|ADN85595.1|IND-NAGPUR-2006(CIIMS-C)-E1 protein Chikungunya virus	-	-	-	-	-	E
76	2006 IND-RJ gi|ABN04194.1|IND-06-RJ1	S	I	M	A	I	E
77	2006 IND-TN gi|ABN04196.1|India TN1 2006	S	I	M	A	I	E
78	2006 JAPAN gi|BAH97931.1|JAPAN-2006-SL11131-structural polyprotein Chikungunya virus	S	I	M	A	I	E
79	2006 JAPAN gi|BAH97933.1|JAPAN-2006-SL10571-structural polyprotein Chikungunya virus	S	I	M	A	I	E
80	2006 MALAYSIA gi|ACD93573.1|Malaysia BP021 2006	.	.	.	.	.	.
81	2006 MALAYSIA gi|CAX63317.2|MALAYSIA-2006-MY/06/37348-structural polyprotein Chikungunya virus	-	-	-	-	-	.
82	2006 MALAYSIA gi|CAX63318.1|MALAYSIA-2006-MY/06/37350-structural polyprotein Chikungunya virus	-	-	-	-	-	.
83	2006 MAURITIUS gi|ABJ98544.1|MAU D570 06	.	.	M	A	I	E
84	2006 MAURITIUS gi|ACD93608.1|TM25 (Mauritius)	.	.	M	A	I	E
85	2006 MAURITIUS gi|ACV88657.1|MAURITIUS-2006-BNI-CHIKV-structural polyprotein Chikungunya virus	.	.	M	A	I	E
86	2006 RAW UNION gi|ABF22748.1|RU IMT6466 06	-	-	-	-	-	E
87	2006 RAW UNION gi|ABF22749.1|RU IMT6470 06	-	-	-	-	-	E
88	2006 RAW UNION gi|ABF22750.1|RU IMT6382 06	-	-	-	-	-	E
89	2006 SINGAPORE gi|ACY66841.1|SINGAPORE-2006-0611aTw-structural polyprotein Chikungunya virus	S	I	M	A	I	E
90	2006 SRILANKA gi|ACZ72971.1|SRILANKA-2006-SL15649-structural polyprotein Chikungunya virus	S	I	M	.	I	E
91	2006 USA CALIFORNIA gi|ADG95901.1|USACALIFORNIA-2006-IMPORTED FROM GUJRAT-structural polyprotein Chikungunya virus	S	I	M	A	I	E
92	2007 GABON gi|ACB58723.1|GABON-2007-structural polyprotein Chikungunya virus	-	-	-	.	.	.
93	2007 GABON gi|AFM38219.1|Gabon-2007-structural polyprotein partial Chikungunya virus	-	-	-	-	-	-
94	2007 GABON gi|AFM38221.1|Gabon-2007-structural polyprotein partial Chikungunya virus	-	-	-	-	-	-
95	2007 IND-AP gi|AEJ18139.1|IND-AP-2007-envelope protein E1 Chikungunya virus	-	-	-	-	-	E
96	2007 IND-AP gi|AEJ18140.1|IND-AP-2007-E2 protein Chikungunya virus	-	-	M	A	-	-
97	2007 INDIA gi|ACA51887.1|India 2007	-	-	-	-	-	E
98	2007 INDIA gi|ABX71054.1|India 2007	-	-	-	-	-	E
99	2007 INDIA gi|ABX71055.1|India 2007	-	-	-	-	-	E
100	2007 INDIA gi|ABX71056.1|India 2007	-	-	-	-	-	E
101	2007 INDIA gi|ABX71057.1|India 2007	-	-	-	-	-	E
102	2007 INDIA gi|ABX71058.1|India 2007	-	-	-	-	-	E
103	2007 INDIA gi|ACA51885.1|India 2007	-	-	-	-	-	E
104	2007 INDIA gi|AEQ59652.1|IND-2007-envelope glycoprotein 1 Chikungunya virus	-	-	-	-	-	-
105	2007 INDIA gi|AEQ59653.1|IND-2007-envelope glycoprotein 1 Chikungunya virus	-	-	-	-	-	-
106	2007 INDIA gi|AEQ59654.1|IND-2007-envelope glycoprotein 1 Chikungunya virus	-	-	-	-	-	-
107	2007 INDIA gi|AEQ59655.1|IND-2007-envelope glycoprotein 1 Chikungunya virus	-	-	-	-	-	-
108	2007 INDIA gi|AEQ59656.1|IND-2007-envelope glycoprotein 1 Chikungunya virus	-	-	-	-	-	-
109	2007 INDIA gi|AEQ59657.1|IND-2007-envelope glycoprotein 1 Chikungunya virus	-	-	-	-	-	-
110	2007 IND-KR gi|ACA81773.1|IND-KL-2007-DRDE-07-structural polyprotein Chikungunya virus	S	.	M	A	I	E
111	2007 IND-KR gi|ACM09921.1|IND-KR-2007-IND-KR52-structural polyprotein Chikungunya virus	S	I	M	A	I	E
112	2007 IND-KR gi|ACY25942.1|IND-2007-RGCB80/KL07-structural polyprotein Chikungunya virus	S	I	M	A	I	E
113	2007 IND-KR gi|ACY25944.1|IND-2007-RGCB120/KL07-structural polyprotein Chikungunya virus	S	I	M	A	I	E
114	2007 INDONESIA gi|ACY66830.1|INDONESIA-2007-0712aTW-structural polyprotein Chikungunya virus	.	.	.	.	.	.
115	2007 INDONESIA gi|ACY66831.1|INDONESIA-2007-0712bTw-structural polyprotein Chikungunya virus	.	.	.	.	.	.
116	2007 INDONESIA gi|ACY66843.1|INDONESIA-2007-0607aTw-structural polyprotein Chikungunya virus	.	.	.	.	.	.
117	2007 IND-SGPGI gi|ACE75875.1|SGPGI/2007/01	-	-	-	-	-	E
118	2007 IND-SGPGI gi|ACE75876.1|SGPGI/2007/02	-	-	-	-	-	E
119	2007 IND-SGPGI gi|ACE75877.1|SGPGI/2007/05	-	-	-	-	-	E
120	2007 ITALY gi|ABX38965.1| ITA07-RA1	S	I	M	A	I	E
121	2007 MOURITIUS gi|ABU93705.1|Wuerzburg (Mauritius)	.	.	M	A	I	E
122	2007 SRILANKA gi|ADC53731.1|SRI-LANKA-2007-LKRGCH2707structural polyprotein Chikungunya virus	S	I	M	A	I	E
123	2007 SRILANKA gi|ADC53732.1|SRI-LANKA-2007-LKRGCH1507-structural polyprotein Chikungunya virus	S	I	M	A	I	E
124	2007 SRILANKA gi|ADG95909.1|SRI LANKA-2007-SL-CR-structural polyprotein Chikungunya virus	S	I	M	A	I	E
125	2007 SRILANKA gi|ADG95913.1|SRI-LANKA-2007-SL-CK1-structural polyprotein Chikungunya virus	S	I	M	A	I	E

	Amino acid position of structral polyprotein						1
				6	7	7	0
		2	2	3	1	5	9
**Sr. No**	**Strain Details**	3	7	7	1	6	3
**1**	**1953 South Africa gi|AAN05102.2|S27 (African prototype)**	**P**	**V**	**T**	**V**	**V**	**D**
126	2008 AUSTRALIA gi|ACS45303.1|AUSTRALIA-2008-Mal2008-structural polyprotein Chikungunya virus	S	I	M	A	I	E
127	2008 BANGLADESH gi|ACY66845.1|BANGLADESH-2008-0810aTw-structural polyprotein Chikungunya virus	S	.	M	A	I	E
128	2008 CHINA gi|ACZ98835.1|CHINA-2008-FD080008- structural polyprotein Chikungunya virus	S	I	M	A	I	E
129	2008 CHINA gi|ACZ98837.1|CHINA-2008-SD08Pan-structural polyprotein Chikungunya virus	S	.	M	A	I	E
130	2008 CHINA gi|ACZ98839.1|CHINA-2008-FD080178-structural polyprotein Chikungunya virus	S	I	M	A	I	E
131	2008 CHINA gi|ACZ98841.1|CHINA-2008-FD080231-structural polyprotein Chikungunya virus	S	I	M	A	I	E
132	2008 INDIA gi|ACY66838.1|IND-2008-0812cTw-structural polyprotein Chikungunya virus	S	I	M	A	I	E
133	2008 INDIA gi|AEK21840.1|India-2008-structural polyprotein Chikungunya virus	S	.	M	A	I	E
134	2008 IND-KA gi|ADN85586.1|IND-KA-2008 E1 protein Chikungunya virus	-	-	-	-	-	E
135	2008 IND-KA gi|ADN85587.1|IND-KA-2008(DRDE-08)-E1 protein Chikungunya virus	-	-	-	-	-	E
136	2008 IND-KA gi|ADN85588.1|IND-KA-2008(DRDE-08)-E1 protein Chikungunya virus	-	-	-	-	-	E
137	2008 IND-KA gi|ADN85589.1|IND-KA-2008(DRDE-08)-E1 protein Chikungunya virus	-	-	-	-	-	E
138	2008 IND-KA gi|ADN85590.1|IND-KA-2008(DRDE-08)-E1 protein Chikungunya virus	-	-	-	-	-	E
139	2008 IND-KA gi|ADN85591.1|IND-KA-2008(DRDE-08)-E1 protein Chikungunya virus	-	-	-	-	-	E
140	2008 IND-KA gi|ADN85592.1|IND-KA-2008(DRDE-08)-E1 protein Chikungunya virus	-	-	-	-	-	E
141	2008 IND-KA gi|ADN85593.1|IND-KA-2008(DRDE-08)-E1 protein Chikungunya virus	-	-	-	-	-	E
142	2008 IND-KR gi|ACY25946.1|IND-2008-RGCB355/KL08-structural polyprotein Chikungunya virus	S	I	M	A	I	E
143	2008 IND-KR gi|ACY25948.1|IND-2008-RGCB356/KL08-structural polyprotein Chikungunya virus	S	.	M	A	I	E
144	2008 INDONESIA gi|ACY66832.1|INDONESIA-2008-0802aTw-structural polyprotein Chikungunya virus	.	.	.	.	.	.
145	2008 INDONESIA gi|ACY66833.1|INDONESIA-2008-0802aTW-structural polyprotein Chikungunya virus	.	.	.	.	.	.
146	2008 INDONESIA gi|ACY66834.1|INDONESIA-2008-0806aTW-structural polyprotein Chikungunya virus	.	.	.	.	.	.
147	2008 INDONESIA gi|ACY66835.1|INDONESIA-2008-0811aTW-structural polyprotein Chikungunya virus	.	.	.	.	.	.
148	2008 MALAYSIA gi|ACY66836.1|MALASIA-2008-0812aTW-structural polyprotein Chikungunya virus	S	I	M	A	I	E
149	2008 MALAYSIA gi|ACY66837.1|MALASIA-2008-0812bTW-structural polyprotein Chikungunya virus	S	I	M	A	I	E
150	2008 MALAYSIA gi|ACY66847.1|Malaysia 0810bTw 2008	S	I	M	A	I	E
151	2008 MALAYSIA gi|ACY66847.1|MALAYSIA-2008-0810aTw-structural polyprotein Chikungunya virus	S	I	M	A	I	E
152	2008 MALAYSIA gi|ADC84390.1|MALAYSIA-2008-envelope protein E1 Chikungunya virus	-	-	-	-	-	E
153	2008 MALAYSIA gi|CAX63319.2|MALAYSIA-2008-MY/08/065-structural polyprotein Chikungunya virus	-	-	-	-	-	E
154	2008 MALAYSIA gi|CAX63320.1|MALAYSIA-2008-MY/08/066-structural polyprotein Chikungunya virus	-	-	-	-	-	E
155	2008 MALAYSIA gi|CAX63321.1|MALAYSIA-2008-MY/08/068-structural polyprotein Chikungunya virus	-	-	-	-	-	E
156	2008 MALAYSIA gi|CAX63322.1|MALAYSIA-2008-MY/08/2868-structural polyprotein Chikungunya virus	-	-	-	-	-	E
157	2008 MALAYSIA gi|CAX63323.2|MALAYSIA-2008-MY/08/2844-structural polyprotein Chikungunya virus	-	-	-	-	-	E
158	2008 MALAYSIA gi|CAX63324.1|MALAYSIA-2008-MY/08/6000-structural polyprotein Chikungunya virus	-	-	-	-	-	E
159	2008 MALAYSIA gi|CAX63325.1|MALAYSIA-2008-MY/08/5369-structural polyprotein Chikungunya virus	-	-	-	-	-	E
160	2008 MALAYSIA gi|CAX63326.1|MALAYSIA-2008-MY/08/7913-structural polyprotein Chikungunya virus	-	-	-	-	-	E
161	2008 MALAYSIA gi|CAX63327.1|MALAYSIA-2008-MY/08/0539-structural polyprotein Chikungunya virus	-	-	-	-	-	E
162	2008 MALAYSIA gi|CAX63328.1|MALAYSIA-2008-MY/08/2561-structural polyprotein Chikungunya virus	-	-	-	-	-	E
163	2008 MALAYSIA gi|CAX63329.1|MALAYSIA-2008-MY/08/6008-structural polyprotein Chikungunya virus	-	-	-	-	-	E
164	2008 SINGAPORE gi|ADC53733.1|SINGAPURE-2008-SGEHICHD93508-structural polyprotein Chikungunya virus	S	I	M	A	I	E
165	2008 SINGAPORE gi|ADC53734.1|SINGAPURE-2008-SGEHICHD421708-structural polyprotein Chikungunya virus	S	I	M	A	I	E
166	2008 SINGAPORE gi|ADC53735.1|SINGAPURE-2008-SGEHICHS422308-structural polyprotein Chikungunya virus	S	I	M	A	I	E
167	2008 SINGAPORE gi|ADC53736.1|SINGAPURE-2008-SGEHICHS422808-structural polyprotein Chikungunya virus	S	I	M	A	I	E
168	2008 SINGAPORE gi|ADC53737.1|SINGAPURE-2008-SGEHICHS424108-structural polyprotein Chikungunya virus	S	I	M	A	I	E
169	2008 SINGAPORE gi|ADC53738.1|SINGAPURE-2008-SGEHICHS425208-structural polyprotein Chikungunya virus	S	I	M	A	I	E
170	2008 SINGAPORE gi|ADC53739.1|SINGAPURE-2008-SGEHICHD96808-structural polyprotein Chikungunya virus	S	I	M	A	I	E
171	2008 SINGAPORE gi|ADC53740.1|SINGAPURE-2008-SGEHICHT077808-structural polyprotein Chikungunya virus	S	I	M	A	I	E
172	2008 SINGAPORE gi|ADC53741.1|SINGAPURE-2008-SGEHICHD122508-structural polyprotein Chikungunya virus	S	I	M	A	I	E
173	2008 SINGAPORE gi|ADC53742.1|SINGAPURE-2008-SGEHICHS277108- structural polyprotein Chikungunya virus	S	I	M	A	I	E
174	2008 SINGAPORE gi|ADC53743.1|SINGAPURE-2008-SGEHICHD13508-structural polyprotein Chikungunya virus	S	I	M	A	I	E
175	2008 SRILANKA gi|ACY09939.1|SRILANKA-2008-LK(PB)CH1008-structural polyprotein Chikungunya virus	S	I	M	A	I	E
176	2008 SRILANKA gi|ACY09940.1|SRILANKA-2008-LK(PB)CH1608-structural polyprotein Chikungunya virus	S	I	M	A	I	E
177	2008 SRILANKA gi|ACY09941.1|SRILANKA-2008-LK(PB)CH3008-structural polyprotein Chikungunya virus	S	.	M	A	I	E
178	2008 SRILANKA gi|ACY09943.1|SRILANKA-2008-LK(PB)CH5308-structural polyprotein Chikungunya virus	S	I	M	A	I	E
179	2008 SRILANKA gi|ACY09945.1|SRILANKA-2008-LK(PB)CH5808-structural polyprotein Chikungunya virus	S	I	M	A	I	E
180	2008 SRILANKA gi|ACY09946.1|SRILANKA-2008-LK(PB)CH4408-structural polyprotein Chikungunya virus	S	.	M	A	I	E
181	2008 SRILANKA gi|ACY09948.1|SRILANKA-2008-LK(PB)CH6708-structural polyprotein Chikungunya virus	S	.	M	A	I	E
182	2008 SRILANKA gi|ACY09949.1|SRILANKA-2008-LK(PB)CH7708-structural polyprotein Chikungunya virus	S	I	M	A	I	E
183	2008 SRILANKA gi|ACY09951.1|SRILANKA-2008-LK(EH)CH17708-structural polyprotein Chikungunya virus	S	.	M	A	I	E
184	2008 SRILANKA gi|ACY09952.1|SRILANKA-2008-LK(EH)CH18608-structural polyprotein Chikungunya virus	S	.	M	A	I	E
185	2008 SRILANKA gi|ACY09953.1|SRILANKA-2008-LK(EH)CH20108-structural polyprotein Chikungunya virus	S	I	M	A	I	E
186	2008 SRILANKA gi|ACZ93023.2|SRILANKA-2008-LK(PB)chik3408-structural polyprotein Chikungunya virus	S	I	M	A	I	E
187	2008 SRILANKA gi|ACZ93024.2|SRILANKA-2008-LK(PB)chik6008-structural polyprotein Chikungunya virus	S	I	M	A	I	E
188	2008 SRILANKA gi|ACZ93025.2|SRILANKA-2008-LK(EH)chik19708-structural polyprotein Chikungunya virus	S	I	M	A	I	E
189	2008 SRILANKA gi|ADC53730.1|SRILANKA-2008-LKRGCH13908-structural polyprotein Chikungunya virus	S	.	M	A	I	E

	Amino acid position of structral polyprotein						1
				6	7	7	0
		2	2	3	1	5	9
**Sr. No**	**Strain Details**	3	7	7	1	6	3
**1**	**1953 South Africa gi|AAN05102.2|S27 (African prototype)**	**P**	**V**	**T**	**V**	**V**	**D**
190	2008 THIALAND gi|ADJ19190.1|THIALAND-2008-CU-Chikstructural polyprotein Chikungunya virus	S	I	M	A	I	E
191	2009 IND-AP gi|ACT10814.1|IND-AP-2009-envelope protein Chikungunya virus	-	-	-	-	-	E
192	2009 IND-AP gi|ACT10815.1|IND-AP-2009-envelope protein Chikungunya virus	-	-	-	-	-	E
193	2009 IND-AP gi|ACT10816.1|IND-AP-2009-envelope protein Chikungunya virus	-	-	-	-	-	E
194	2009 IND-AP gi|ACT10817.1|IND-AP-2009-envelope protein Chikungunya virus	-	-	-	-	-	E
195	2009 IND-AP gi|ADZ04936.1|IND-AP-2009-structural polyprotein Chikungunya virus	S	I	M	A	I	E
196	2009 INDIA gi|AEE60797.1|IND-2009-structural polyprotein Chikungunya virus	S	I	M	A	I	E
197	2009 INDIA gi|AEK21842.1|India-2009 structural polyprotein Chikungunya virus	S	I	M	A	I	E
198	2009 IND-KA gi|AEX92839.1|IND-KA-2009-structural polyprotein partial Chikungunya virus	-	-	-	-	-	E
199	2009 IND-KA gi|AEX92840.1|IND-KA-2009-structural polyprotein partial Chikungunya virus	-	-	-	-	-	E
200	2009 IND-KA gi|AEX92841.1|IND-KA-2009-structural polyprotein partial Chikungunya virus	-	-	-	-	-	E
201	2009 IND-KA gi|AEX92842.1|IND-KA-2009-structural polyprotein partial Chikungunya virus	-	-	-	-	-	E
202	2009 IND-KA gi|AEX92843.1|IND-KA-2009-structural polyprotein partial Chikungunya virus	-	-	-	-	-	E
203	2009 IND-KA gi|AEX92844.1|IND-KA-2009-structural polyprotein partial Chikungunya virus	-	-	-	-	-	E
204	2009 IND-KA gi|AEX92845.1|IND-KA-2009-structural polyprotein partial Chikungunya virus	-	-	-	-	-	E
205	2009 IND-KA gi|AEX92846.1|IND-KA-2009-structural polyprotein partial Chikungunya virus	-	-	-	-	-	E
206	2009 IND-KA gi|AEX92847.1|IND-KA-2009-structural polyprotein partial Chikungunya virus	-	-	-	-	-	E
207	2009 IND-KR gi|ADW19890.1|IND-KL-2009-RGCB696-E2 envelope protein Chikungunya virus	-	-	M	A	-	-
208	2009 IND-KR gi|ADW19891.1|IND-KL-2009-RGCB699-E2 envelope protein Chikungunya virus	-	-	M	A	-	-
209	2009 IND-KR gi|ADW19892.1|IND-2009-RGCB-msq1-chlym09-E2 envelope protein Chikungunya virus	-	-	M	A	-	-
210	2009 IND-KR gi|ADW19893.1|IND-2009-RGCB-msq1-01vna09-E2 envelope protein Chikungunya virus	-	-	M	A	-	-
211	2009 IND-KR gi|ADW19894.1|IND-2009-RGCB-msq1-Bpr09-E2 envelope protein Chikungunya virus	-	-	M	A	-	-
212	2009 MALAYSIA gi|ACY66839.1|MALASIA-2009-0901aTW-structural polyprotein Chikungunya virus	S	I	M	A	I	E
213	2009 THIALAND gi|ADI88515.1|THAILAND-2009-CU-Chik-structural polyprotein Chikungunya virus	S	I	M	A	I	E
214	2009 THIALAND gi|ADJ19188.1|THAILAND-2009-CU-Chik009-structural polyprotein Chikungunya virus	S	I	M	A	I	E
215	2009 THIALAND gi|ADJ19192.1|THAILAND-2009-CU-Chik-structural polyprotein Chikungunya virus	S	I	M	A	I	E
216	2009 THIALAND gi|ADK24722.1|THAILAND-2009-CU-Chik-structural polyprotein Chikungunya virus	S	I	M	A	I	E
217	2009 THIALAND gi|ADQ37313.1|SINGAPUR-2009-structural polyprotein Chikungunya virus	S	I	M	A	I	E
218	2009 THIALAND gi|ADQ37314.1|Thailand-2009-structural polyprotein Chikungunya virus	S	I	M	A	I	E
219	2010 CENTRAL AFRICAN REPBLIC gi|ADG95899.1|CENTRAL AFRICA REPUBLIC()2010-structural polyprotein Chikungunya virus	.	.	.	.	.	.
220	2010 CHINA gi|AEK31253.1|CHINA-2010-structural polyprotein Chikungunya virus	S	I	M	A	I	E
221	2010 CHINA gi|AEK31255.1|CHINA-2010-structural polyprotein Chikungunya virus	S	I	M	A	I	E
222	2010 CHINA gi|AEK31257.1|CHINA-2010-structural polyprotein Chikungunya virus	S	I	M	A	I	E
223	2010 CHINA gi|AEK31259.1|CHINA-2010-structural polyprotein Chikungunya virus	S	I	M	A	I	E
224	2010 CHINA gi|AEX25334.1|CHINA-2010-structural polyprotein Chikungunya virus	S	I	M	A	I	E
225	2010 CHINA gi|AEX25336.1|CHINA-2010-structural polyprotein Chikungunya virus	S	I	M	A	I	E
226	2010 CHINA gi|AEX25338.1|CHINA-2010-structural polyprotein Chikungunya virus	S	I	M	A	I	E
227	2010 CHINA gi|AEX25340.1|CHINA-2010-structural polyprotein Chikungunya virus	S	I	M	A	I	E
228	2010 CHINA gi|AEX25342.1|CHINA-2010-structural polyprotein Chikungunya virus	S	I	M	A	I	E
229	2010 CHINA gi|AEX25344.1|CHINA-2010-structural polyprotein Chikungunya virus	S	I	M	A	I	E
230	2010 CHINA gi|AEX25346.1|CHINA-2010-structural polyprotein Chikungunya virus	S	I	M	A	I	E
231	2010 CHINA gi|AEX25348.1|CHINA-2010-structural polyprotein partial Chikungunya virus	S	I	M	A	I	E
232	2010 CHINA gi|AFD61558.1|China-2010-structural polyprotein Chikungunya virus	.	.	.	.	.	.
233	2010 CHINA gi|AFP43244.1|China-2010 structural polyprotein Chikungunya virus	S	I	M	A	I	E
234	2010 FRANCE gi|CCA61128.1|FRANCE-2010-structural polyprotein E2-6K-E1 region Chikungunya virus	-	-	M	A	I	E
235	2010 FRANCE gi|CCA61129.1|FRANCE-2010-structural polyprotein E2-6K-E1 region Chikungunya virus	-	-	M	A	I	E
236	2010 FRANCE gi|CCA61130.1|FRANCE-2010-structural polyprotein E2-6K-E1 region Chikungunya virus	-	-	M	A	I	E
237	2010 FRANCE gi|CCA61131.1|FRANCE-2009-structural polyprotein E2-6K-E1 region Chikungunya virus	-	-	.	.	.	.
238	2010 GABON gi|AFM38220.1|Gabon-2010-structural polyprotein partial Chikungunya virus	-	-	-	-	-	-
239	2010 IND-AP gi|AEJ18138.1|IND-AP-2010-E2 protein Chikungunya virus	-	-	M	A	-	-
240	2010 IND-DEL gi|AEV42237.1|IND-10-DEL12 2010	-	-	-	-	-	E
241	2010 IND-DEL gi|AEV42239.1|IND-10-DEL106 2010	-	-	-	-	-	E
242	2010 IND-DEL gi|AEV42241.1|IND-10-DEL110 2010	-	-	-	-	-	E
243	2010 IND-DEL gi|AEV42243.1|IND-10-DEL123 2010	-	-	-	-	-	E
244	2010 IND-DEL gi|AEV42245.1|IND-10-DEL100 2010	-	-	-	-	-	E
245	2010 IND-DEL gi|AEV42247.1|IND-10-DEL19 2010	-	-	-	-	-	E
246	2010 IND-DEL gi|AEV42249.1|IND-10-DEL15 2010	-	-	-	-	-	E
247	2010 INDIA gi|AEE60792.1|IND-2010-structural polyprotein Chikungunya virus	S	I	M	A	I	E
248	2010 INDIA gi|AEE60793.1|IND-2010-structural polyprotein Chikungunya virus	S	I	M	A	I	E
249	2010 IND-KA gi|AEX92848.1|IND-KA-2010-structural polyprotein partial Chikungunya virus	-	-	-	-	-	E
250	2010 IND-KA gi|AEX92849.1|IND-KA-2010-structural polyprotein partial Chikungunya virus	-	-	-	-	-	E
251	2010 IND-ORISSA gi|AFA41483.1|22/Mosquito/RMRC/2010	-	-	-	-	-	E
252	2010 IND-ORISSA gi|AFA41484.1|23/Mosquito/RMRC/2010	-	-	-	-	-	E
253	2010 IND-ORISSA gi|AFA43355.1|10/RMRC/2010	-	-	-	-	-	E

	**Amino acid position of structral polyprotein**						1
				6	7	7	0
		2	2	3	1	5	9
**Sr. No**	**Strain Details**	3	7	7	1	6	3
**1**	**1953 South Africa gi|AAN05102.2|S27 (African prototype)**	**P**	**V**	**T**	**V**	**V**	**D**
254	2010 IND-ORISSA gi|AFA43356.1|11/RMRC/2010	-	-	-	-	-	E
255	2010 IND-ORISSA gi|AFA43357.1|12/RMRC/2010	-	-	-	-	-	E
256	2010 IND-ORISSA gi|AFA43358.1|13/RMRC/2010	-	-	-	-	-	E
257	2010 IND-ORISSA gi|AFA43359.1|14/RMRC/2010	-	-	-	-	-	E
258	2010 IND-ORISSA gi|AFA43360.1|15/RMRC/2010	-	-	-	-	-	E
259	2010 IND-ORISSA gi|AFA43361.1|16/RMRC/2010	-	-	-	-	-	E
260	2010 IND-ORISSA gi|AFA43362.1|17/RMRC/2010	-	-	-	-	-	E
261	2010 IND-ORISSA gi|AFA43363.1|18/RMRC/2010	-	-	-	-	-	E
262	2010 IND-ORISSA gi|AFA43364.1|19/RMRC/2010	-	-	-	-	-	E
263	2010 IND-ORISSA gi|AFA43365.1|20/RMRC/2010	-	-	-	-	-	E
264	2010 IND-ORISSA gi|AFA43366.1|21/RMRC/2010	-	-	-	-	-	E
265	2010 SENEGAL gi|ADG95919.1|SENEGAL-()2010-IPD/A SH 2807-structural polyprotein Chikungunya virus	.	.	.	.	A	.
266	2011 COMBODIA gi|AFM35612.1|Combodia-2011-structural polyprotein Chikungunya virus	S	I	M	A	I	E
267	2011 COMBODIA gi|AFM35614.1|Combodia-2011-structural polyprotein Chikungunya virus	S	I	M	A	I	E
268	2011 COMBODIA gi|AFM35616.1|Combodia-2011-structural polyprotein Chikungunya virus	S	I	M	A	I	E
269	2011 COMBODIA gi|AFM35618.1|Combodia-2011-structural polyprotein Chikungunya virus	S	I	M	A	I	E
270	2011 COMBODIA gi|AFM35620.1|Combodia-2011-structural polyprotein Chikungunya virus	S	I	M	A	I	E
271	2011 COMBODIA gi|AFM35622.1|Combodia-2011-structural polyprotein Chikungunya virus	S	I	M	A	I	E
272	2011 COMBODIA gi|AFM35624.1|Combodia-2011-structural polyprotein partial Chikungunya virus	S	I	M	A	I	E
273	2011 COMBODIA gi|AFM35626.1|Combodia-2011-structural polyprotein Chikungunya virus	S	I	M	A	I	E
