# Supplementary material for: A Novel 2006 Indian Outbreak Strain of Chikungunya Virus Exhibits Different Pattern of Infection as Compared to Prototype Strain
Source: PLoS One. 2014 Jan 20;9(1):e85714. doi: 10.1371/journal.pone.0085714 (PMC3896419; doi:10.1371/journal.pone.0085714)
Supplement: Table S3 — Details of the CHIKV Non-Structural protein sequences of different global strains along with accession numbers (n = 157) used in this study. Amino acid position of consistent mutations present in this region is shown along with alignment. (PDF) [file pone.0085714.s006.pdf]

Table S3. Details of the CHIKV Non-Structural protein sequences of different global strains along with accession numbers (n=157) used in this study. Amino acid position of consistent mutations present in this region is shown along with alignment.

	**Amino acid position of Non-structral polyprotein**				1	1	1	1	1	2
		1	3	5	3	5	6	8	9	1
		2	7	8	2	5	7	0	3	1
**Sr. No**	**Strain Details**	8	6	9	8	0	0	4	8	7
**1**	**1953 SOUTHAFRICA gi|AAN05101.1|S27-African prototype AF369024 1 nonstructural polyprotein Chikungunya viru**	**T**	**T**	**S**	**A**	**Y**	**T**	**P**	**T**	**T**
2	1953 SOUTHAFRICA gi|NP 690588.1|S27-African prototype nonstructural polyprotein Chikungunya virus	.	.	.	.	.	.	.	.	.
3	1953 SENEGAL gi|ADG95918.1|IPD/A SH 2807 nonstructural polyprotein Chikungunya virus	A	.	.	.	.	.	.	.	.
4	1953 TANZANIA gi|ADG95931.1|Ross low-psg nonstructural polyprotein Chikungunya virus	.	.	.	.	.	.	.	.	.
5	1954 CENTRAL AFRICAN REPUBLIC gi|ADG95898.1|CAR256 nonstructural polyprotein Chikungunya virus	.	.	.	.	.	.	.	.	.
6	1956 SOUTH AFRICA gi|ADG95896.1|Vereeniging nonstructural polyprotein Chikungunya virus	.	.	.	.	.	.	.	.	.
7	1958 THIALAND gi|ADG95929.1|TH35 nonstructural polyprotein Chikungunya virus	.	.	.	.	.	.	.	.	.
8	1960 CONGO gi|ADG95927.1|LSFS nonstructural polyprotein Chikungunya virus	.	.	.	.	.	.	.	I	.
9	1962 ANGOLA gi|ADG95955.1|Angola M2022 nonstructural polyprotein Chikungunya virus	.	.	.	.	.	.	.	.	.
10	1963 INDIA gi|ADG95916.1|I-634029 nonstructural polyprotein Chikungunya virus	.	.	.	.	.	A	.	.	.
11	1963 INDIA gi|ADG95935.1|Gibbs 63-263 nonstructural polyprotein Chikungunya virus	.	.	.	.	.	A	.	.	.
12	1963 IND-WB gi|ABN04199.1|IND-63-WB1 nonstructural polyprotein Chikungunya virus	.	.	.	.	.	A	.	.	.
13	1963 SENEGAL gi|ADG95951.1|A301 nonstructural polyprotein Chikungunya virus	.	.	.	.	.	.	.	.	.
14	1964 NIGERIA gi|ADG95884.1|IbH35 nonstructural polyprotein Chikungunya virus	A	.	.	.	.	.	.	.	.
15	1966 SENEGAL gi|ADG95882.1|PM2951 nonstructural polyprotein Chikungunya virus	A	.	.	.	.	.	.	.	.
16	1966 SENEGAL gi|ADG95941.1|SH 3013 nonstructural polyprotein Chikungunya virus	A	.	.	.	.	.	.	.	.
17	1973 INDIA gi|ADG95888.1|PO731460 nonstructural polyprotein Chikungunya virus	.	.	.	.	.	A	.	.	.
18	1973 IND-MH gi|ABN04201.1|IND-73-MH5 nonstructural polyprotein Chikungunya virus	.	.	.	.	.	A	.	.	.
19	1975 THIALAND gi|ADG95937.1|1455-75 nonstructural polyprotein Chikungunya virus	.	.	.	.	.	.	.	.	.
20	1976 SOUTH AFRICA gi|ADG95920.1|AR 18211 nonstructural polyprotein Chikungunya virus	.	.	.	.	.	.	.	.	.
21	1978 CENTRAL AFRICAN REPUBLIC gi|ADG95953.1|HB78 nonstructural polyprotein Chikungunya virus	.	.	.	.	.	.	.	.	.
22	1978 THIALAND gi|ADG95925.1|3412-78 nonstructural polyprotein Chikungunya virus	.	.	.	.	.	.	.	.	.
23	1979 SENEGAL gi|ADG95939.1|ArD 30237 nonstructural polyprotein Chikungunya virus	A	.	.	.	.	.	.	.	.
24	1981 COTE DIVOIRE gi|ADG95945.1|ArA 2657 nonstructural polyprotein Chikungunya virus	A	.	.	.	.	.	.	.	.
25	1982 UGANDA gi|ADG95933.1|UgAg4155 nonstructural polyprotein Chikungunya virus	.	.	.	.	.	.	.	.	.
26	1983 INDONESIA gi|ADG95894.1|JKT23574 nonstructural polyprotein Chikungunya virus	.	.	.	.	.	.	.	.	.
27	1983 SENEGAL gi|AAU43880.1|37997 nonstructural polyprotein Chikungunya virus	A	.	.	.	.	.	.	.	.
28	1984 CENTRAL AFRICAN REPUBLIC gi|ADG95880.1|DakAr B 16878 nonstructural polyprotein Chikungunya virus	.	.	.	.	.	.	.	.	.
29	1985 INDONESIA gi|ADG95905.1|RSU1 nonstructural polyprotein Chikungunya virus	.	.	.	.	.	.	.	.	.
30	1985 PHILIPPINES gi|ADG95892.1|PhH15483 nonstructural polyprotein Chikungunya virus	.	.	.	.	.	.	.	.	.
31	1985 PHILIPPINES gi|ADG95910.1|Hu/85/NR/001 nonstructural polyprotein Chikungunya virus	.	.	.	.	.	.	.	.	.
32	1986 INDIA gi|ADG95922.1|ALSA-1 nonstructural polyprotein Chikungunya virus	.	.	.	.	.	-	.	.	.
33	1988 THIALAND gi|ADG95890.1|6441-88 nonstructural polyprotein Chikungunya virus	.	.	.	.	.	.	.	.	.
34	1993 COTE D IVOIRE gi|ADG95949.1|ArA 30548 nonstructural polyprotein Chikungunya virus	A	.	.	.	.	.	.	.	.
35	1993 SENEGAL gi|ADG95947.1|ArD 93229 nonstructural polyprotein Chikungunya virus	A	.	.	.	.	.	.	.	.
36	1995 THIALAND gi|ADG95886.1|SV0444-95 nonstructural polyprotein Chikungunya virus	.	.	.	.	.	.	.	.	.
37	1995 THIALAND gi|ADG95903.1|CO392-95 nonstructural polyprotein Chikungunya virus	.	.	.	.	.	.	.	.	.
38	1995 THIALAND gi|ADG95914.1|K0146-95 nonstructural polyprotein Chikungunya virus	.	.	.	.	.	.	.	.	.
39	2000 IND-MH gi|ABN04197.1|IND-00-MH4 nonstructural polyprotein Chikungunya virus	.	.	.	.	.	.	.	.	.
40	2001 COMBODIA gi|AFM35613.1|V1024386 KH11 PVH nonstructural polyprotein Chikungunya virus	K	M	N	V	H	I	S	A	A
41	2004-05 INDIAN OCEAN gi|AEG64807.1|Com25 nonstructural polyprotein Chikungunya virus	.	.	N	V	H	I	S	A	A
42	2004-05 INDIAN OCEAN gi|AEG64808.1|COMJ nonstructural polyprotein Chikungunya virus	.	.	N	V	H	I	S	A	A
43	2004-05 INDIAN OCEAN gi|AEG64809.1|Com125 nonstructural polyprotein Chikungunya virus	.	.	N	V	H	I	S	A	A
44	2004-05 INDIAN OCEAN gi|AEG64810.1|KPA15 nonstructural polyprotein Chikungunya virus	.	.	N	V	H	I	S	A	A
45	2004-05 INDIAN OCEAN gi|AEG64811.1|Lamu33 nonstructural polyprotein Chikungunya virus	.	M	N	V	H	I	S	A	A
46	2005 LAREUNION gi|ABJ98543.1|La Reunion 06-021 non-structural polyprotein Chikungunya virus	.	.	N	V	H	I	S	A	A
47	2005 SENEGAL gi|ADG95943.1|HD 180760 nonstructural polyprotein Chikungunya virus	A	.	.	.	.	.	.	.	.
48	2006 CALIFORNIA gi|ADG95900.1|DHS4263-Calif AB nonstructural polyprotein Chikungunya virus	K	M	N	V	H	I	S	A	A
49	2006 IND-AP gi|ABN04187.1|IND-06-AP3 nonstructural polyprotein Chikungunya virus	K	M	N	V	H	I	S	A	A
**50**	**2006 IND-AP gi|ABP88821.1|DRDE-06 DRDEHydISW06 nonstructural polyprotein Chikungunya virus**	**K**	**M**	**N**	**V**	**H**	**I**	**S**	**A**	**A**
51	2006 IND-GUJ gi|ACM09906.1|IND-GJ52 nonstructural polyprotein Chikungunya virus	K	M	N	V	H	I	S	A	A
52	2006 IND-GUJ gi|ACM09912.1|IND-GJ53 nonstructural polyprotein Chikungunya virus	K	M	N	V	H	I	S	A	A
53	2006 IND-GUJ gi|AEA10290.1|IND-06-Guj nonstructural polyprotein Chikungunya virus	K	M	N	V	H	I	S	A	A
54	2006 IND-GUJgi|ACM09910.1|IND-GJ51 nonstructural polyprotein Chikungunya virus	K	M	N	V	H	I	S	A	A
55	2006 INDIA-KA gi|ACM09918.1|IND-KA51 nonstructural polyprotein Chikungunya virus	K	M	N	V	H	I	S	A	A
56	2006 IND-KA gi|ABN04189.1|IND-06-KA15 nonstructural polyprotein Chikungunya virus	K	M	N	V	H	I	S	A	A
57	2006 IND-KA gi|ACM09908.1|IND-KA52 nonstructural polyprotein Chikungunya virus	K	M	N	V	H	I	S	A	A
58	2006 IND-KL gi|ACY25937.1|RGCB03/KL06 nonstructural polyprotein Chikungunya virus	K	M	N	V	H	I	S	A	A
59	2006 IND-KL gi|ACY25939.1|RGCB05/KL06 nonstructural polyprotein Chikungunya virus	K	M	N	V	H	I	S	A	A
60	2006 IND-KR gi|ACM09914.1|IND-KR51 nonstructural polyprotein Chikungunya virus	K	M	N	V	H	I	S	A	A
61	2006 IND-MH gi|ABN04191.1|IND-06-MH2 nonstructural polyprotein Chikungunya virus	K	M	N	V	H	I	S	A	A
62	2006 IND-MH gi|ACM09916.1|IND-MH51 nonstructural polyprotein Chikungunya virus	K	M	N	V	H	I	S	A	A
63	2006 IND-RAJ gi|ACD93609.1|CHIK31 nonstructural polyprotein Chikungunya virus	K	M	N	V	H	I	S	A	A
64	2006 IND-RJ gi|ABN04193.1|IND-06-RJ1 nonstructural polyprotein Chikungunya virus	K	M	N	V	H	I	S	A	A
65	2006 IND-TN gi|ABN04195.1|IND-06-TN1 nonstructural polyprotein Chikungunya virus	K	M	N	V	H	I	S	A	A
66	2006 JAPAN gi|BAH97930.1|SL11131 PATIENT RETURN FROM SRILANKA nonstructural polyprotein Chikungunya virus	K	M	N	V	H	I	S	A	A
67	2006 JAPAN gi|BAH97932.1|SL10571 PATIENT RETURN FROM SRILANKA nonstructural polyprotein Chikungunya virus	K	M	N	V	H	I	S	A	A
68	2006 LAW RE UNION gi|ABX40005.1|LR2006 OPY1 nonstructural polyprotein Cloning vector pCHIKV-LR ic	.	.	N	V	H	I	S	A	A
69	2006 LAW RE UNION gi|ABX40009.1| LR2006 OPY1 nonstructural polyprotein Cloning vector pCHIK-LR 5GFP	.	.	N	V	H	I	S	A	A
70	2006 LAW RE UNION gi|ABX40010.1|LR2006 OPY1 nonstructural polyprotein Cloning vector pCHIK-37997ic	A	.	.	.	.	.	.	.	.
71	2006 LR2006 OPY1 gi|ABX40012.1|LR2006 OPY1 nonstructural polyprotein Cloning vector pCHIK-37997-5GFP	A	.	.	.	.	.	.	.	.
72	2006 MALAYSIA gi|ABJ98543.1|Malaysia BP002 non-structural polyprotein Chikungunya virus	.	.	N	V	H	I	S	A	A
73	2006 MAURITIUS gi|ABJ98543.1|D570/06 non-structural polyprotein Chikungunya virus	.	.	N	V	H	I	S	A	A
74	2006 MAURITIUS gi|ACD93607.1|TM25 nonstructural polyprotein Chikungunya virus	.	.	N	V	H	I	S	A	A
75	2006 REUNION gi|ABD95937.2|LR2006 OPY1 nonstructural polyprotein Chikungunya virus	.	.	N	V	H	I	S	A	A
76	2006 SINGAPORE gi|ACY66840.1|0611aTw nonstructural polyprotein Chikungunya virus	K	M	N	V	H	I	S	A	A
77	2006 SRILANKA gi|ACY09944.1|LK(PB)CH5808 nonstructural polyprotein Chikungunya virus	K	M	N	V	H	I	S	A	A
78	2006 SRILANKA gi|ACZ72970.1|SL15649 nonstructural polyprotein Chikungunya virus	K	M	N	V	H	I	S	A	A
79	2006-07 THIALAND gi|ABO38820.1|AF15561 nonstructural polyprotein Chikungunya virus	.	.	.	.	.	.	.	.	.

	Amino acid position of Non-structral polyprotein				1	1	1	1	1	2
		1	3	5	3	5	6	8	9	1
		2	7	8	2	5	7	0	3	1
**Sr. No**	**Strain Details**	8	6	9	8	0	0	4	8	7
**1**	**1953 SOUTHAFRICA gi|AAN05101.1|S27-African prototype AF369024 1 nonstructural polyprotein Chikungunya virus**	**T**	**T**	**S**	**A**	**Y**	**T**	**P**	**T**	**T**
80	2007 CHINA gi|ADJ67656.1|Xinjiang nonstructural polyprotein Chikungunya virus	K	-	-	-	-	-	-	-	-
81	2007 IND-KL gi|ACA81772.1|DRDE-07 nonstructural polyprotein Chikungunya virus	K	M	N	V	H	I	S	A	A
82	2007 IND-KL gi|ACY25941.1|RGCB80/KL07 nonstructural polyprotein Chikungunya virus	K	M	N	V	H	I	S	A	A
83	2007 IND-KL gi|ACY25943.1|RGCB120/KL07 nonstructural polyprotein Chikungunya virus	K	M	N	V	H	I	S	A	A
84	2007 IND-KR gi|ACM09920.1|IND-KR52 nonstructural polyprotein Chikungunya virus	K	M	N	V	H	I	S	A	A
85	2007 INDONESIA gi|ACY66842.1|0706aTw nonstructural polyprotein Chikungunya virus	.	.	.	.	H	.	.	.	.
86	2007 ITALY gi|ABX38963.1|ITA07-RA1 nonstructural polyprotein long variant Chikungunya virus	K	M	N	V	H	I	S	A	A
87	2007 ITALY gi|ABX38964.1|ITA07-RA1 nonstructural polyprotein short variant Chikungunya virus	K	M	N	V	H	I	S	-	-
88	2007 MAURITIUS gi|ABJ98543.1|Wuerzburg 2007 non-structural polyprotein Chikungunya virus	.	.	N	V	H	I	S	A	A
89	2007 SRILANKA gi|ACS29295.2|LKMTCH2707 nonstructural polyprotein Chikungunya virus	K	M	N	V	H	I	S	A	A
90	2007 SRILANKA gi|ACS29296.2|LKRGCH1507 nonstructural polyprotein Chikungunya virus	K	M	N	V	H	I	S	A	A
91	2007 SRILANKA gi|ADG95908.1|SL-CR 3 nonstructural polyprotein Chikungunya virus	K	M	N	V	H	I	S	A	A
92	2007 SRILANKA gi|ADG95912.1|SL-CK1 nonstructural polyprotein Chikungunya virus	K	M	N	V	H	I	S	A	A
93	2007 USA gi|ABO10016.1|TSI-GSD-218 nonstructural polyprotein Chikungunya virus	.	.	.	.	.	.	.	.	.
94	2007 USA gi|ABO38822.1|TSI-GSD-218-VR1 nonstructural polyprotein Chikungunya virus	.	.	.	.	.	.	.	.	.
95	2008 BANGLADESH gi|ACY66844.1|0810aTw nonstructural polyprotein Chikungunya virus	K	M	N	V	H	I	S	A	A
96	2008 CHINA gi|ACZ98834.1|FD080008 nonstructural polyprotein Chikungunya virus	K	M	N	V	H	I	S	A	A
97	2008 CHINA gi|ACZ98836.1|SD08Pan nonstructural polyprotein Chikungunya virus	K	M	N	V	H	I	S	A	A
98	2008 CHINA gi|ACZ98838.1|FD080178 nonstructural polyprotein Chikungunya virus	K	M	N	V	H	I	S	A	A
99	2008 CHINA gi|ACZ98840.1|FD080231 nonstructural polyprotein Chikungunya virus	K	M	N	V	H	I	S	A	A
100	2008 IND-APgi|AEE60907.1|CHIKNSP4-SVU08 nonstructural polyprotein Chikungunya virus	-	-	-	-	-	-	S	A	A
101	2008 INDIA gi|AEK21841.1|CHIK-KDP08 nonstructural polyprotein Chikungunya virus	-	-	-	-	-	-	S	A	A
102	2008 IND-KL gi|ACY25945.1|RGCB355/KL08 nonstructural polyprotein Chikungunya virus	K	M	N	V	H	I	S	A	A
103	2008 IND-KL gi|ACY25947.1|RGCB356/KL08 nonstructural polyprotein Chikungunya virus	K	M	N	V	H	I	S	A	A
104	2008 MALAYSIA gi|ACY66846.1|0810bTw nonstructural polyprotein Chikungunya virus	K	M	N	V	H	I	S	A	A
105	2008 SINGAPORE gi|ACS29298.2|SGEHICHD93508 nonstructural polyprotein Chikungunya virus	K	M	N	V	H	I	S	A	A
106	2008 SINGAPORE gi|ACS29299.2|SGEHICHS421708 nonstructural polyprotein Chikungunya virus	K	M	N	V	H	I	S	A	A
107	2008 SINGAPORE gi|ACS29300.2|SGEHICHS422308 nonstructural polyprotein Chikungunya virus	K	M	N	V	H	I	S	A	A
108	2008 SINGAPORE gi|ACS29301.2|SGEHICHS422808 nonstructural polyprotein Chikungunya virus	K	M	N	V	H	I	S	A	A
109	2008 SINGAPORE gi|ACS29311.2|SGEHICHS424108 nonstructural polyprotein Chikungunya virus	K	M	N	V	H	I	S	A	A
110	2008 SINGAPORE gi|ACS29313.2|SGEHICHS425208 nonstructural polyprotein Chikungunya virus	K	M	N	V	H	I	S	A	A
111	2008 SINGAPORE gi|ACS29331.2|SGEHICHD96808 nonstructural polyprotein Chikungunya virus	K	M	N	V	H	I	S	A	A
112	2008 SINGAPORE gi|ACS29352.2|SGEHICHT077808 nonstructural polyprotein Chikungunya virus	K	M	N	V	H	I	S	A	A
113	2008 SINGAPORE gi|ACS29370.2|SGEHICHD122508 nonstructural polyprotein Chikungunya virus	K	M	N	V	H	I	S	A	A
114	2008 SINGAPORE gi|ACS29378.2|SGEHICHS277108 nonstructural polyprotein Chikungunya virus	K	M	N	V	H	I	S	.	A
115	2008 SINGAPORE gi|ACS29379.2|SGEHICHD13508 nonstructural polyprotein Chikungunya virus	K	M	N	V	H	I	S	.	A
116	2008 SRILANKA gi|ACS29294.2|LKEHCH13908 nonstructural polyprotein Chikungunya virus	K	M	N	V	H	I	S	A	A
117	2008 SRILANKA gi|ACT35031.1|LK(PB)CH1008 nonstructural polyprotein Chikungunya virus	K	M	N	V	H	I	S	A	A
118	2008 SRILANKA gi|ACT35032.1|LK(PB)CH1608 nonstructural polyprotein Chikungunya virus	K	M	N	V	H	I	S	A	A
119	2008 SRILANKA gi|ACT35035.1|LK(PB)CH3008 nonstructural polyprotein Chikungunya virus	K	M	N	V	H	I	S	A	A
120	2008 SRILANKA gi|ACT35048.1|LK(EH)CH4408 nonstructural polyprotein Chikungunya virus	K	M	N	V	H	I	S	A	A
121	2008 SRILANKA gi|ACT35060.1|LK(EH)CH7708 nonstructural polyprotein Chikungunya virus	K	M	N	V	H	I	S	A	A
122	2008 SRILANKA gi|ACT35078.1|LK(EH)CH18608 nonstructural polyprotein Chikungunya virus	K	M	N	V	H	I	S	A	A
123	2008 SRILANKA gi|ACT35082.1|LK(EH)CH20108 nonstructural polyprotein Chikungunya virus	K	M	N	V	H	I	S	A	A
124	2008 SRILANKA gi|ACY09942.1|LK(PB)CH5308 nonstructural polyprotein Chikungunya virus	K	M	N	V	H	I	S	A	A
125	2008 SRILANKA gi|ACY09947.1|LK(EH)CH6708 nonstructural polyprotein Chikungunya virus	K	M	N	V	H	I	S	A	A
126	2008 SRILANKA gi|ACY09950.1|LK(EH)CH17708 nonstructural polyprotein Chikungunya virus	K	M	N	V	H	I	S	A	A
127	2008 SRILANKA gi|ADC53744.1|LK(EH)chik19708 nonstructural polyprotein Chikungunya virus	K	M	N	V	H	I	S	A	A
128	2008 SRILANKA gi|ADC53745.1|LK(PB)chik6008 nonstructural polyprotein Chikungunya virus	K	M	N	V	H	I	S	A	A
129	2008 SRILANKA gi|ADC53746.1|LK(PB)chik3408 nonstructural polyprotein Chikungunya virus	K	M	N	V	H	I	S	A	A
130	2008 THIALAND gi|ADJ19189.1|CU-Chik10 nonstructural polyprotein Chikungunya virus	K	M	N	V	H	I	S	A	A
131	2009 IND-AP gi|ADH43195.1|CHIK-CTR09 nonstructural protein 1 Chikungunya virus	K	M	-	-	-	-	-	-	-
132	2009 INDIA gi|AEK21843.1|CHIK-KDP09 nonstructural polyprotein Chikungunya virus	-	-	-	-	-	-	S	A	A
133	2009 THIALAND gi|ADI88516.1|CU-Chik661 truncated nonstructural polyprotein Chikungunya virus	K	M	N	V	H	I	S	-	-
134	2009 THIALAND gi|ADJ19187.1|CU-Chik009 nonstructural polyprotein Chikungunya virus	K	M	N	V	H	I	S	A	A
135	2009 THIALAND gi|ADJ19191.1|CU-Chik683 nonstructural polyprotein Chikungunya virus	K	M	N	V	H	I	S	A	A
136	2009 THIALAND gi|ADK24721.1|CU-Chik OBF nonstructural polyprotein Chikungunya virus	K	M	N	V	H	I	S	A	A
137	2010 CHINA gi|AEK31252.1|GD115 nonstructural polyprotein Chikungunya virus	K	M	N	V	H	I	S	A	A
138	2010 CHINA gi|AEK31254.1|GD113 nonstructural polyprotein Chikungunya virus	K	M	N	V	H	I	S	A	A
139	2010 CHINA gi|AEK31256.1|GD139 nonstructural polyprotein Chikungunya virus	K	M	N	V	H	I	S	A	A
140	2010 CHINA gi|AEK31258.1|GD134 nonstructural polyprotein Chikungunya virus	K	M	N	V	H	I	S	A	A
141	2010 CHINA gi|AEX25333.1|DG891 nonstructural polyprotein partial Chikungunya virus	K	M	N	V	H	I	S	A	A
142	2010 CHINA gi|AEX25335.1|DG892 nonstructural polyprotein partial Chikungunya virus	K	M	N	V	H	I	S	A	A
143	2010 CHINA gi|AEX25337.1|DG893 nonstructural polyprotein partial Chikungunya virus	K	M	N	V	H	I	S	A	A
144	2010 CHINA gi|AEX25339.1|DG894 nonstructural polyprotein partial Chikungunya virus	K	M	N	V	H	I	S	A	A
145	2010 CHINA gi|AEX25341.1|DG895 nonstructural polyprotein partial Chikungunya virus	K	M	N	V	H	I	S	A	A
146	2010 CHINA gi|AEX25343.1|GZ0991 nonstructural polyprotein Chikungunya virus	K	M	N	V	H	I	S	A	A
147	2010 CHINA gi|AEX25345.1|GZ1029 nonstructural polyprotein Chikungunya virus	K	M	N	V	H	I	S	A	A
148	2010 CHINA gi|AEX25347.1|GZ3626 nonstructural polyprotein Chikungunya virus	K	M	N	V	H	I	S	A	A
149	2010 CHINA gi|AFD61557.1|CHI2010 nonstructural polyprotein Chikungunya virus	.	.	.	.	.	I	.	.	.
150	2010 CHINA gi|AFP43243.1|GD05/2010 nonstructural polyprotein Chikungunya virus	K	M	N	V	H	I	S	A	A
151	2011 CAMBODIA gi|AFM35625.1|V1024310 KH11 PVH nonstructural polyprotein Chikungunya virus	K	M	N	V	H	I	S	A	A
152	2011 COMBODIA gi|AFM35611.1|V1024306 KH11 PVH nonstructural polyprotein Chikungunya virus	K	M	N	V	H	I	S	A	A
153	2011 COMBODIA gi|AFM35615.1|V1024310 KH11 PVH nonstructural polyprotein Chikungunya virus	K	M	N	V	H	I	S	A	A
154	2011 COMBODIA gi|AFM35617.1|V1024311 KH11 PVH nonstructural polyprotein Chikungunya virus	K	M	N	V	H	I	S	A	A
155	2011 COMBODIA gi|AFM35619.1|V1024313 KH11 PVH nonstructural polyprotein Chikungunya virus	K	M	N	V	H	I	S	A	A
156	2011 COMBODIA gi|AFM35621.1|V1024314 KH11 PVH nonstructural polyprotein Chikungunya virus	K	M	N	V	H	I	S	A	A
157	2011 COMBODIA gi|AFM35623.1|V1024308 KH11 PVH nonstructural polyprotein Chikungunya virus	K	M	N	V	H	I	S	A	A
