# Supplementary material for: A Novel 2006 Indian Outbreak Strain of Chikungunya Virus Exhibits Different Pattern of Infection as Compared to Prototype Strain
Source: PLoS One. 2014 Jan 20;9(1):e85714. doi: 10.1371/journal.pone.0085714 (PMC3896419; doi:10.1371/journal.pone.0085714)
Supplement: Table S4 — Showing the alignment of E1 structural protein sequence from amino acid position 217 (1026 aa for polyprotein) to 236 (1045 aa for polyprotein) of different global strains used in this study. The position of E1-A226V (1035aa for polyprotein) mutation has been highlighted to show the occurrence of this mutation. (PDF) [file pone.0085714.s007.pdf]

Table S4: Showing the alignment of E1 structural protein sequence from amino acid position 217 (1026 aa for polyprotein) to 236 (1045 aa for polyprotein) of different global strains used in this study. The position of E1-A226V (1035aa for polyprotein) mutation has been highlighted to show the occurrence of this mutation.

		E1 Protein Position											**226**										
		Structural polyprotein position		1026	1027	1028	1029	1030	1031	1032	1033	1034	**1035**	1036	1037	1038	1039	1040	1041	1042	1043	1044	1045
**Sr. No**	**Year**	**Country**	**Acc no**																				
**1**	**1952**	**Africa**	**AAN05102.2**	T	Q	L	V	L	Q	R	P	A	A	G	T	V	H	V	P	Y	S	Q	A
2	1953	TANZANIA	ADG95932.1	.	.	.	.	.	.	.	.	.	.	.	.	.	.	.	.	.	.	.	.
3	1956	AFRICA	ADG95897.1	.	.	.	.	.	.	.	.	.	.	.	.	.	.	.	.	.	.	.	.
4	1958	THAILAND	ADG95930.1	.	.	.	.	.	.	.	.	S	.	.	.	.	.	.	.	.	.	.	.
5	1960	CONGO	ADG95928.1	.	.	.	.	.	.	.	.	.	.	.	.	.	.	.	.	.	.	.	.
6	1962	ANGOLA	ADG95956.1	.	.	.	.	.	.	.	.	.	.	.	.	.	.	.	.	.	.	.	.
7	1963	INDIA	ADG95917.1	.	.	.	.	.	.	.	.	S	.	.	.	.	.	.	.	.	.	.	.
8	1963	INDIA	ADG95936.1	.	.	.	.	.	.	.	.	S	.	.	.	.	.	.	.	.	.	.	.
9	1963	SENEGAL	ADG95952.1	.	.	.	.	.	.	.	.	.	.	.	.	.	.	.	.	.	.	.	.
10	1964	NIGERIA	ADG95885.1	.	.	.	.	.	.	.	.	.	.	.	.	.	.	.	.	.	.	.	.
11	1965	NIGERIA	ADG95924.1	.	.	.	.	.	.	.	.	.	.	.	.	.	.	.	.	.	.	.	.
12	1966	SENEGAL	ADG95883.1	.	.	.	.	.	.	.	.	.	.	.	.	.	.	.	.	.	.	.	.
13	1966	SENEGAL	ADG95907.1	.	.	.	.	.	.	.	.	.	.	.	.	.	.	.	.	.	.	.	.
14	1966	SENEGAL	ADG95942.1	.	.	.	.	.	.	.	.	.	.	.	.	.	.	.	.	.	.	.	.
15	1973	INDIA	ADG95889.1	.	.	.	.	.	.	.	.	S	.	.	.	.	.	.	.	.	.	.	.
16	1975	THIALAND	ADG95938.1	.	.	.	.	.	.	.	.	S	.	.	.	.	.	.	.	.	.	.	.
17	1976	AFRICA	ADG95902.1	.	.	.	.	.	.	.	.	.	.	.	.	.	.	.	.	.	.	.	.
18	1976	AFRICA	ADG95921.1	.	.	.	.	.	.	.	.	.	.	.	.	.	.	.	.	.	.	.	.
19	1978	REPUBLIC	ADG95954.1	.	.	.	.	.	.	.	.	.	.	.	.	.	.	.	.	.	.	.	.
20	1978	THIALAND	ADG95926.1	.	.	.	.	.	.	.	.	S	.	.	.	.	.	.	.	.	.	.	.
21	1979	SENEGAL	ADG95940.1	.	.	.	.	.	.	.	.	.	.	.	.	.	.	.	.	.	.	.	.
22	1981	DIVOIRE	ADG95946.1	.	.	.	.	.	.	.	.	.	.	.	.	.	.	.	.	.	.	.	.
23	1982	UGNADA	ADG95934.1	.	.	.	.	.	.	.	.	.	.	.	.	.	.	.	.	.	.	.	.
24	1983	INDONESIA	ADG95895.1	.	.	.	.	.	.	.	.	S	.	.	.	.	.	.	.	.	.	.	.
25	1984	REPUBLIC	ADG95881.1	.	.	.	.	.	.	.	.	.	.	.	.	.	.	.	.	.	.	.	.
26	1985	INDONESIA	ADG95906.1	.	.	.	.	.	.	.	.	S	.	.	.	.	.	.	.	.	.	.	.
27	1985	PHILIPPINNES	ADG95893.1	.	.	.	.	.	.	.	.	S	.	.	.	.	.	.	.	.	.	.	.
28	1985	PHILIPPINNES	ADG95911.1	.	.	.	.	.	.	.	.	S	.	.	.	.	.	.	.	.	.	.	.
29	1986	IND	ADG95923.1	.	.	.	.	.	.	.	.	.	.	.	.	.	.	.	.	.	.	.	.
30	1988	THIALAND	ADG95891.1	.	.	.	.	.	.	.	.	S	.	.	.	.	.	.	.	.	.	.	.
31	1993	DIVOIRE	ADG95950.1	.	.	.	.	.	.	.	.	.	.	.	.	.	.	.	.	.	.	.	.
32	1993	SENEGAL	ADG95948.1	.	.	.	.	.	.	.	.	.	.	.	.	.	.	.	.	.	.	.	.
33	1995	THIALAND	ADG95887.1	.	.	.	.	.	.	.	.	S	.	.	.	.	.	.	.	.	.	.	.
34	1995	THIALAND	ADG95904.1	.	.	.	.	.	.	.	.	S	.	.	.	.	.	.	.	.	.	.	.
35	1995	THIALAND	ADG95915.1	.	.	.	.	.	.	.	.	S	.	.	.	.	.	.	.	.	.	.	.
36	2000	IND-MH	ABN04198.1	.	.	.	.	.	.	.	.	.	.	.	.	.	.	.	.	.	.	.	.
37	2001	JAPAN	YP 006491243.1	-	-	-	-	-	-	-	-	-	-	-	-	-	-	-	-	-	-	-	-
38	2005	UNION	CAJ90470.1	.	.	.	.	.	.	.	.	.	.	.	.	.	.	.	.	.	.	.	.
39	2005	SENEGAL	ADG95944.1	.	.	.	.	.	.	.	.	.	.	.	.	.	.	.	.	.	.	.	.
40	2005	Seychelles	CAJ90473.1	.	.	.	.	.	.	.	.	.	.	.	.	.	.	.	.	.	.	.	.

41	2006	CAMROON	ABN04115.1	.	.	.	.	.	.	.	.	.	V	.	.	.	.	.	.	.	.	.	.
42	2006	IND-AP	ABN04188.1	.	.	.	.	.	.	.	.	.	.	.	.	.	.	.	.	.	.	.	.
43	2006	IND-GUJ	ACM09907.1	.	.	.	.	.	.	.	.	.	.	.	.	.	.	.	.	.	.	.	.
44	2006	IND-GUJ	ACM09911.1	.	.	.	.	.	.	.	.	.	.	.	.	.	.	.	.	.	.	.	.
45	2006	IND-GUJ	ACM09913.1	.	.	.	.	.	.	.	.	.	.	.	.	.	.	.	.	.	.	.	.
46	2006	IND-GUJ	AEA10291.1	.	.	.	.	.	.	.	.	.	.	.	.	.	.	.	.	.	.	.	.
47	2006	INDIA	ABO40448.1	.	.	.	.	.	.	.	.	.	.	.	.	.	.	.	.	.	.	.	.
48	2006	INDIA	ABO40451.1	.	.	.	.	.	.	.	.	.	.	.	.	.	.	.	.	.	.	.	.
49	2006	INDIA	ABO40453.1	.	.	.	.	.	.	.	.	.	.	.	.	.	.	.	.	.	.	.	.
50	2006	INDIA	ABP88822.1	.	.	.	.	.	.	.	.	.	.	.	.	.	.	.	.	.	.	.	.
51	2006	INDIA	ABO40446.1	.	.	.	.	.	.	.	.	.	.	.	.	.	.	.	.	.	.	.	.
52	2006	INDIA	ABO40447.1	.	.	.	.	.	.	.	.	.	.	.	.	.	.	.	.	.	.	.	.
53	2006	INDIA	ABO40449.1	.	.	.	.	.	.	.	.	.	.	.	.	.	.	.	.	.	.	.	.
54	2006	INDIA	ABO40450.1	.	.	.	.	.	.	.	.	.	.	.	.	.	.	.	.	.	.	.	.
55	2006	INDIA	ABO40452.1	.	.	.	.	.	.	.	.	.	.	.	.	.	.	.	.	.	.	.	.
56	2006	INDIA	AEE60791.1	.	.	.	.	.	.	.	.	.	.	.	.	.	.	.	.	.	.	.	.
57	2006	INDIA	AEE60794.1	.	.	.	.	.	.	.	.	.	.	.	.	.	.	.	.	.	.	.	.
58	2006	INDIA	AEE60795.1	.	.	.	.	.	.	.	.	.	.	.	.	.	.	.	.	.	.	.	.
59	2006	INDIA	AEE60796.1	.	.	.	.	.	.	.	.	.	.	.	.	.	.	.	.	.	.	.	.
60	2006	INDIA	AEQ59648.1	-	-	-	-	-	-	-	-	-	-	-	-	-	-	-	-	-	-	-	-
61	2006	INDIA	AEQ59649.1	-	-	-	-	-	-	-	-	-	-	-	-	-	-	-	-	-	-	-	-
62	2006	INDIA	AEQ59650.1	-	-	-	-	-	-	-	-	-	-	-	-	-	-	-	-	-	-	-	-
63	2006	INDIA	AEQ59651.1	-	-	-	-	-	-	-	-	-	-	-	-	-	-	-	-	-	-	-	-
64	2006	IND-KA	ABN04190.1	.	.	.	.	.	.	.	.	.	.	.	.	.	.	.	.	.	.	.	.
65	2006	IND-KA	ACM09909.1	.	.	.	.	.	.	.	.	.	.	.	.	.	.	.	.	.	.	.	.
66	2006	IND-KA	ACM09919.1	.	.	.	.	.	.	.	.	.	.	.	.	.	.	.	.	.	.	.	.
67	2006	IND-KL	ACY25938.1	.	.	.	.	.	.	.	.	.	.	.	.	.	.	.	.	.	.	.	.
68	2006	IND-KL	ACY25940.1	.	.	.	.	.	.	.	.	.	.	.	.	.	.	.	.	.	.	.	.
69	2006	IND-KR	ACM09915.1	.	.	.	.	.	.	.	.	.	.	.	.	.	.	.	.	.	.	.	.
70	2006	IND-MAH	ACM09917.1	.	.	.	.	.	.	.	.	.	.	.	.	.	.	.	.	.	.	.	.
71	2006	IND-MH	ABN04192.1	.	.	.	.	.	.	.	.	.	.	.	.	.	.	.	.	.	.	.	.
72	2006	IND-NAGPUR	ADN85594.1	.	.	.	.	.	.	.	.	.	.	.	.	.	.	.	.	.	.	.	.
73	2006	IND-NAGPUR	ADN85594.1	.	.	.	.	.	.	.	.	.	.	.	.	.	.	.	.	.	.	.	.
74	2006	IND-NAGPUR	ADN85595.1	.	.	.	.	.	.	.	.	.	.	.	.	.	.	.	.	.	.	.	.
75	2006	IND-NAGPUR	ADN85595.1	.	.	.	.	.	.	.	.	.	.	.	.	.	.	.	.	.	.	.	.
76	2006	IND-RJ	ABN04194.1	.	.	.	.	.	.	.	.	.	.	.	.	.	.	.	.	.	.	.	.
77	2006	IND-TN	ABN04196.1	.	.	.	.	.	.	.	.	.	.	.	.	.	.	.	.	.	.	.	.
78	2006	JAPAN	BAH97931.1	.	.	.	.	.	.	.	.	.	.	.	.	.	.	.	.	.	.	.	.
79	2006	JAPAN	BAH97933.1	.	.	.	.	.	.	.	.	.	.	.	.	.	.	.	.	.	.	.	.
80	2006	MALAYSIA	ACD93573.1	.	.	.	.	.	.	.	.	S	.	.	.	.	.	.	.	.	.	.	.
81	2006	MALAYSIA	CAX63317.2	.	.	.	.	.	.	.	.	S	.	.	.	.	.	.	.	.	.	.	.
82	2006	MALAYSIA	CAX63318.1	.	.	.	.	.	.	.	.	S	.	.	.	.	.	.	.	.	.	.	.
83	2006	MAURITIUS	ABJ98544.1	.	.	.	.	.	.	.	.	.	V	.	.	.	.	.	.	.	.	.	.
84	2006	MAURITIUS	ACD93608.1	.	.	.	.	.	.	.	.	.	V	.	.	.	.	.	.	.	.	.	.
85	2006	MAURITIUS	ACV88657.1	.	.	.	.	.	.	.	.	.	V	.	.	.	.	.	.	.	.	.	.
86	2006	UNION	ABF22748.1	.	.	.	.	.	.	.	.	.	V	.	.	.	.	.	.	.	.	.	.
87	2006	UNION	ABF22749.1	.	.	.	.	.	.	.	.	.	V	.	.	.	.	.	.	.	.	.	.
88	2006	UNION	ABF22750.1	.	.	.	.	.	.	.	.	.	V	.	.	.	.	.	.	.	.	.	.
89	2006	SINGAPORE	ACY66841.1	.	.	.	.	.	.	.	.	.	.	.	.	.	.	.	.	.	.	.	.
90	2006	SRILANKA	ACZ72971.1	.	.	.	.	.	.	.	.	.	.	.	.	.	.	.	.	.	.	.	.
91	2006	CALIFORNIA	ADG95901.1	.	.	.	.	.	.	.	.	.	.	.	.	.	.	.	.	.	.	.	.
92	2007	GABON	ACB58723.1	.	.	.	.	.	.	.	.	.	V	.	.	.	.	.	.	.	.	.	.

93	2007	GABON	AFM38219.1	.	.	.	.	.	.	.	.	.	G	-	.	.	.	.	.	.	.	.	.
94	2007	GABON	AFM38221.1	.	.	.	.	.	.	.	.	.	V	.	.	.	.	.	.	.	.	.	.
95	2007	IND-AP	AEJ18139.1	.	.	.	.	.	.	.	.	.	.	.	.	.	.	.	.	.	.	.	.
96	2007	IND-AP	AEJ18140.1	-	-	-	-	-	-	-	-	-	-	-	-	-	-	-	-	-	-	-	-
97	2007	INDIA	ACA51887.1	.	.	.	.	.	.	.	.	.	V	.	.	.	.	.	.	.	.	.	.
98	2007	INDIA	ABX71054.1	.	.	.	.	.	.	.	.	.	V	.	.	.	.	.	.	.	.	.	.
99	2007	INDIA	ABX71055.1	.	.	.	.	.	.	.	.	.	V	.	.	.	.	.	.	.	.	.	.
100	2007	INDIA	ABX71056.1	.	.	.	.	.	.	.	.	.	V	.	.	.	.	.	.	.	.	.	.
101	2007	INDIA	ABX71057.1	.	.	.	.	.	.	.	.	.	V	.	.	.	.	.	.	.	.	.	.
102	2007	INDIA	ABX71058.1	.	.	.	.	.	.	.	.	.	V	.	.	.	.	.	.	.	.	.	.
103	2007	INDIA	ACA51885.1	.	.	.	.	.	.	.	.	.	V	.	.	.	.	.	.	.	.	.	.
104	2007	INDIA	AEQ59652.1	-	-	-	-	-	-	-	-	-	-	-	-	-	-	-	-	-	-	-	-
105	2007	INDIA	AEQ59653.1	-	-	-	-	-	-	-	-	-	-	-	-	-	-	-	-	-	-	-	-
106	2007	INDIA	AEQ59654.1	-	-	-	-	-	-	-	-	-	-	-	-	-	-	-	-	-	-	-	-
107	2007	INDIA	AEQ59655.1	-	-	-	-	-	-	-	-	-	-	-	-	-	-	-	-	-	-	-	-
108	2007	INDIA	AEQ59656.1	-	-	-	-	-	-	-	-	-	-	-	-	-	-	-	-	-	-	-	-
109	2007	INDIA	AEQ59657.1	-	-	-	-	-	-	-	-	-	-	-	-	-	-	-	-	-	-	-	-
110	2007	IND-KR	ACA81773.1	.	.	.	.	.	.	.	.	.	V	.	.	.	.	.	.	.	.	.	.
111	2007	IND-KR	ACM09921.1	.	.	.	.	.	.	.	.	.	V	.	.	.	.	.	.	.	.	.	.
112	2007	IND-KR	ACY25942.1	.	.	.	.	.	.	.	.	.	V	.	.	.	.	.	.	.	.	.	.
113	2007	IND-KR	ACY25944.1	.	.	.	.	.	.	.	.	.	V	.	.	.	.	.	.	.	.	.	.
114	2007	INDONESIA	ACY66830.1	.	.	.	.	.	.	.	.	S	.	.	.	.	.	.	.	.	.	.	.
115	2007	INDONESIA	ACY66831.1	.	.	.	.	.	.	.	.	S	.	.	.	.	.	.	.	.	.	.	.
116	2007	INDONESIA	ACY66843.1	.	.	.	.	.	.	.	.	S	.	.	.	.	.	.	.	.	.	.	.
117	2007	IND-SGPGI	ACE75875.1	.	.	.	.	.	.	.	.	.	.	.	.	.	.	.	.	.	.	.	.
118	2007	IND-SGPGI	ACE75876.1	.	.	.	.	.	.	.	.	.	.	.	.	.	.	.	.	.	.	.	.
119	2007	IND-SGPGI	ACE75877.1	.	.	.	.	.	.	.	.	.	.	.	.	.	.	.	.	.	.	.	.
120	2007	ITALY	ABX38965.1	.	.	.	.	.	.	.	.	.	V	.	.	.	.	.	.	.	.	.	.
121	2007	MOURITIUS	ABU93705.1	.	.	.	.	.	.	.	.	.	V	.	.	.	.	.	.	.	.	.	.
122	2007	SRILANKA	ADC53731.1	.	.	.	.	.	.	.	.	.	.	.	.	.	.	.	.	.	.	.	.
123	2007	SRILANKA	ADC53732.1	.	.	.	.	.	.	.	.	.	.	.	.	.	.	.	.	.	.	.	.
124	2007	SRILANKA	ADG95909.1	.	.	.	.	.	.	.	.	.	.	.	.	.	.	.	.	.	.	.	.
125	2007	SRILANKA	ADG95913.1	.	.	.	.	.	.	.	.	.	.	.	.	.	.	.	.	.	.	.	.
126	2008	AUSTRALIA	ACS45303.1	.	.	.	.	.	.	.	.	.	V	.	.	.	.	.	.	.	.	.	.
127	2008	BANGLADESH	ACY66845.1	.	.	.	.	.	.	.	.	.	V	.	.	.	.	.	.	.	.	.	.
128	2008	CHINA	ACZ98835.1	.	.	.	.	.	.	.	.	.	.	.	.	.	.	.	.	.	.	.	.
129	2008	CHINA	ACZ98837.1	.	.	.	.	.	.	.	.	.	.	.	.	.	.	.	.	.	.	.	.
130	2008	CHINA	ACZ98839.1	.	.	.	.	.	.	.	.	.	V	.	.	.	.	.	.	.	.	.	.
131	2008	CHINA	ACZ98841.1	.	.	.	.	.	.	.	.	.	V	.	.	.	.	.	.	.	.	.	.
132	2008	INDIA	ACY66838.1	.	.	.	.	.	.	.	.	.	.	.	.	.	.	.	.	.	.	.	.
133	2008	INDIA	AEK21840.1	.	.	.	.	.	.	.	.	.	.	.	.	.	.	.	.	.	.	.	.
134	2008	IND-KA	ADN85586.1	.	.	.	.	.	.	.	.	.	V	.	.	.	.	.	.	.	.	.	.
135	2008	IND-KA	ADN85587.1	.	.	.	.	.	.	.	.	.	V	.	.	.	.	.	.	.	.	.	.
136	2008	IND-KA	ADN85588.1	.	.	.	.	.	.	.	.	.	V	.	.	.	.	.	.	.	.	.	.
137	2008	IND-KA	ADN85589.1	.	.	.	.	.	.	.	.	.	V	.	.	.	.	.	.	.	.	.	.
138	2008	IND-KA	ADN85590.1	.	.	.	.	.	.	.	.	.	V	.	.	.	.	.	.	.	.	.	.
139	2008	IND-KA	ADN85591.1	.	.	.	.	.	.	.	.	.	V	.	.	.	.	.	.	.	.	.	.
140	2008	IND-KA	ADN85592.1	.	.	.	.	.	.	.	.	.	V	.	.	.	.	.	.	.	.	.	.
141	2008	IND-KA	ADN85593.1	.	.	.	.	.	.	.	.	.	V	.	.	.	.	.	.	.	.	.	.
142	2008	IND-KR	ACY25946.1	.	.	.	.	.	.	.	.	.	V	.	.	.	.	.	.	.	.	.	.
143	2008	IND-KR	ACY25948.1	.	.	.	.	.	.	.	.	.	V	.	.	.	.	.	.	.	.	.	.
144	2008	INDONESIA	ACY66832.1	.	.	.	.	.	.	.	.	S	.	.	.	.	.	.	.	.	.	.	.

145	2008	INDONESIA	ACY66833.1	.	.	.	.	.	.	.	.	S	.	.	.	.	.	.	.	.	.	.	.
146	2008	INDONESIA	ACY66834.1	.	.	.	.	.	.	.	.	S	.	.	.	.	.	.	.	.	.	.	.
147	2008	INDONESIA	ACY66835.1	.	.	.	.	.	.	.	.	S	.	.	.	.	.	.	.	.	.	.	.
148	2008	MALAYSIA	ACY66836.1	.	.	.	.	.	.	.	.	.	V	.	.	.	.	.	.	.	.	.	.
149	2008	MALAYSIA	ACY66837.1	.	.	.	.	.	.	.	.	.	V	.	.	.	.	.	.	.	.	.	.
150	2008	MALAYSIA	ACY66847.1	.	.	.	.	.	.	.	.	.	V	.	.	.	.	.	.	.	.	.	.
151	2008	MALAYSIA	ACY66847.1	.	.	.	.	.	.	.	.	.	V	.	.	.	.	.	.	.	.	.	.
152	2008	MALAYSIA	ADC84390.1	.	.	.	.	.	.	.	.	.	V	.	.	.	.	.	.	.	.	.	.
153	2008	MALAYSIA	CAX63319.2	.	.	.	.	.	.	.	.	.	V	.	.	.	.	.	.	.	.	.	.
154	2008	MALAYSIA	CAX63320.1	.	.	.	.	.	.	.	.	.	V	.	.	.	.	.	.	.	.	.	.
155	2008	MALAYSIA	CAX63321.1	.	.	.	.	.	.	.	.	.	V	.	.	.	.	.	.	.	.	.	.
156	2008	MALAYSIA	CAX63322.1	.	.	.	.	.	.	.	.	.	V	.	.	.	.	.	.	.	.	.	.
157	2008	MALAYSIA	CAX63323.2	.	.	.	.	.	.	.	.	.	V	.	.	.	.	.	.	.	.	.	.
158	2008	MALAYSIA	CAX63324.1	.	.	.	.	.	.	.	.	.	V	.	.	.	.	.	.	.	.	.	.
159	2008	MALAYSIA	CAX63325.1	.	.	.	.	.	.	.	.	.	V	.	.	.	.	.	.	.	.	.	.
160	2008	MALAYSIA	CAX63326.1	.	.	.	.	.	.	.	.	.	V	.	.	.	.	.	.	.	.	.	.
161	2008	MALAYSIA	CAX63327.1	.	.	.	.	.	.	.	.	.	V	.	.	.	.	.	.	.	.	.	.
162	2008	MALAYSIA	CAX63328.1	.	.	.	.	.	.	.	.	.	V	.	.	.	.	.	.	.	.	.	.
163	2008	MALAYSIA	CAX63329.1	.	.	.	.	.	.	.	.	.	V	.	.	.	.	.	.	.	.	.	.
164	2008	SINGAPORE	ADC53733.1	.	.	.	.	.	.	.	.	.	V	.	.	.	.	.	.	.	.	.	.
165	2008	SINGAPORE	ADC53734.1	.	.	.	.	.	.	.	.	.	V	.	.	.	.	.	.	.	.	.	.
166	2008	SINGAPORE	ADC53735.1	.	.	.	.	.	.	.	.	.	V	.	.	.	.	.	.	.	.	.	.
167	2008	SINGAPORE	ADC53736.1	.	.	.	.	.	.	.	.	.	V	.	.	.	.	.	.	.	.	.	.
168	2008	SINGAPORE	ADC53737.1	.	.	.	.	.	.	.	.	.	V	.	.	.	.	.	.	.	.	.	.
169	2008	SINGAPORE	ADC53738.1	.	.	.	.	.	.	.	.	.	V	.	.	.	.	.	.	.	.	.	.
170	2008	SINGAPORE	ADC53739.1	.	.	.	.	.	.	.	.	.	V	.	.	.	.	.	.	.	.	.	.
171	2008	SINGAPORE	ADC53740.1	.	.	.	.	.	.	.	.	.	V	.	.	.	.	.	.	.	.	.	.
172	2008	SINGAPORE	ADC53741.1	.	.	.	.	.	.	.	.	.	V	.	.	.	.	.	.	.	.	.	.
173	2008	SINGAPORE	ADC53742.1	.	.	.	.	.	.	.	.	.	.	.	.	.	.	.	.	.	.	.	.
174	2008	SINGAPORE	ADC53743.1	.	.	.	.	.	.	.	.	.	.	.	.	.	.	.	.	.	.	.	.
175	2008	SRILANKA	ACY09939.1	.	.	.	.	.	.	.	.	.	V	.	.	.	.	.	.	.	.	.	.
176	2008	SRILANKA	ACY09940.1	.	.	.	.	.	.	.	.	.	V	.	.	.	.	.	.	.	.	.	.
177	2008	SRILANKA	ACY09941.1	.	.	.	.	.	.	.	.	.	V	.	.	.	.	.	.	.	.	.	.
178	2008	SRILANKA	ACY09943.1	.	.	.	.	.	.	.	.	.	V	.	.	.	.	.	.	.	.	.	.
179	2008	SRILANKA	ACY09945.1	.	.	.	.	.	.	.	.	.	V	.	.	.	.	.	.	.	.	.	.
180	2008	SRILANKA	ACY09946.1	.	.	.	.	.	.	.	.	.	V	.	.	.	.	.	.	.	.	.	.
181	2008	SRILANKA	ACY09948.1	.	.	.	.	.	.	.	.	.	V	.	.	.	.	.	.	.	.	.	.
182	2008	SRILANKA	ACY09949.1	.	.	.	I	.	.	.	.	.	V	.	.	.	.	.	.	.	.	.	.
183	2008	SRILANKA	ACY09951.1	.	.	.	.	.	.	.	.	.	V	.	.	.	.	.	.	.	.	.	.
184	2008	SRILANKA	ACY09952.1	.	.	.	.	.	.	.	.	.	.	.	.	.	.	.	.	.	.	.	.
185	2008	SRILANKA	ACY09953.1	.	.	.	.	.	.	.	.	.	V	.	.	.	.	.	.	.	.	.	.
186	2008	SRILANKA	ACZ93023.2	.	.	.	.	.	.	.	.	.	V	.	.	.	.	.	.	.	.	.	.
187	2008	SRILANKA	ACZ93024.2	.	.	.	.	.	.	.	.	.	V	.	.	.	.	.	.	.	.	.	.
188	2008	SRILANKA	ACZ93025.2	.	.	.	.	.	.	.	.	.	V	.	.	.	.	.	.	.	.	.	.
189	2008	SRILANKA	ADC53730.1	.	.	.	.	.	.	.	.	.	V	.	.	.	.	.	.	.	.	.	.
190	2008	THIALAND	ADJ19190.1	.	.	.	.	.	.	.	.	.	V	.	.	.	.	.	.	.	.	.	.
191	2009	IND-AP	ACT10814.1	.	.	.	.	.	.	.	.	.	.	.	.	.	.	.	.	.	.	.	.
192	2009	IND-AP	ACT10815.1	.	.	.	.	.	.	.	.	.	.	.	.	.	.	.	.	.	.	.	.
193	2009	IND-AP	ACT10816.1	.	.	.	.	.	.	.	.	.	.	.	.	.	.	.	.	.	.	.	.
194	2009	IND-AP	ACT10817.1	.	.	.	.	.	.	.	.	.	.	.	.	.	.	.	.	.	.	.	.
195	2009	IND-AP	ADZ04936.1	.	.	.	.	.	.	.	.	.	.	.	.	.	.	.	.	.	.	.	.
196	2009	INDIA	AEE60797.1	.	.	.	.	.	.	.	.	.	.	.	.	.	.	.	.	.	.	.	.

197	2009	INDIA	AEK21842.1	.	.	.	.	.	.	.	.	.	.	.	.	.	.	.	.	.	.	.	.
198	2009	IND-KA	AEX92839.1	.	.	.	.	.	.	.	.	.	.	.	.	.	.	.	.	.	.	.	.
199	2009	IND-KA	AEX92840.1	.	.	.	.	.	.	.	.	.	.	.	.	.	.	.	.	.	.	.	.
200	2009	IND-KA	AEX92841.1	.	.	.	.	.	.	.	.	.	.	.	.	.	.	.	.	.	.	.	.
201	2009	IND-KA	AEX92842.1	.	.	.	.	.	.	.	.	.	.	.	.	.	.	.	.	.	.	.	.
202	2009	IND-KA	AEX92843.1	.	.	.	.	.	.	.	.	.	.	.	.	.	.	.	.	.	.	.	.
203	2009	IND-KA	AEX92844.1	.	.	.	.	.	.	.	.	.	.	.	.	.	.	.	.	.	.	.	.
204	2009	IND-KA	AEX92845.1	.	.	.	.	.	.	.	.	.	.	.	.	.	.	.	.	.	.	.	.
205	2009	IND-KA	AEX92846.1	.	.	.	.	.	.	.	.	.	.	.	.	.	.	.	.	.	.	.	.
206	2009	IND-KA	AEX92847.1	.	.	.	.	.	.	.	.	.	.	.	.	.	.	.	.	.	.	.	.
207	2009	IND-KR	ADW19890.1	-	-	-	-	-	-	-	-	-	-	-	-	-	-	-	-	-	-	-	-
208	2009	IND-KR	ADW19891.1	-	-	-	-	-	-	-	-	-	-	-	-	-	-	-	-	-	-	-	-
209	2009	IND-KR	ADW19892.1	-	-	-	-	-	-	-	-	-	-	-	-	-	-	-	-	-	-	-	-
210	2009	IND-KR	ADW19893.1	-	-	-	-	-	-	-	-	-	-	-	-	-	-	-	-	-	-	-	-
211	2009	IND-KR	ADW19894.1	-	-	-	-	-	-	-	-	-	-	-	-	-	-	-	-	-	-	-	-
212	2009	MALAYSIA	ACY66839.1	.	.	.	.	.	.	.	.	.	V	.	.	.	.	.	.	.	.	.	.
213	2009	THIALAND	ADI88515.1	.	.	.	.	.	.	.	.	.	V	.	.	.	.	.	.	.	.	.	.
214	2009	THIALAND	ADJ19188.1	.	.	.	.	.	.	.	.	.	V	.	.	.	.	.	.	.	.	.	.
215	2009	THIALAND	ADJ19192.1	.	.	.	.	.	.	.	.	.	V	.	.	.	.	.	.	.	.	.	.
216	2009	THIALAND	ADK24722.1	.	.	.	.	.	.	.	.	.	V	.	.	.	.	.	.	.	.	.	.
217	2009	THIALAND	ADQ37313.1	.	.	.	.	.	.	.	.	.	V	.	.	.	.	.	.	.	.	.	.
218	2009	THIALAND	ADQ37314.1	.	.	.	.	.	.	.	.	.	V	.	.	.	.	.	.	.	.	.	.
219	2010	REPBLIC	ADG95899.1	.	.	.	.	.	.	.	.	.	.	.	.	.	.	.	.	.	.	.	.
220	2010	CHINA	AEK31253.1	.	.	.	.	.	.	.	.	.	V	.	.	.	.	.	.	.	.	.	.
221	2010	CHINA	AEK31255.1	.	.	.	.	.	.	.	.	.	V	.	.	.	.	.	.	.	.	H	.
222	2010	CHINA	AEK31257.1	.	.	.	.	.	.	.	.	.	V	.	.	.	.	.	.	.	.	.	.
223	2010	CHINA	AEK31259.1	.	.	.	.	.	.	.	.	.	V	.	.	.	.	.	.	.	.	.	.
224	2010	CHINA	AEX25334.1	.	.	.	.	.	.	.	.	.	V	.	.	.	.	.	.	.	.	.	.
225	2010	CHINA	AEX25336.1	.	.	.	.	.	.	.	.	.	V	.	.	.	.	.	.	.	.	.	.
226	2010	CHINA	AEX25338.1	.	.	.	.	.	.	.	.	.	V	.	.	.	.	.	.	.	.	.	.
227	2010	CHINA	AEX25340.1	.	.	.	.	.	.	.	.	.	V	.	.	.	.	.	.	.	.	.	.
228	2010	CHINA	AEX25342.1	.	.	.	.	.	.	.	.	.	V	.	.	.	.	.	.	.	.	.	.
229	2010	CHINA	AEX25344.1	.	.	.	.	.	.	.	.	.	.	.	.	.	.	.	.	.	.	.	.
230	2010	CHINA	AEX25346.1	.	.	.	.	.	.	.	.	.	.	.	.	.	.	.	.	.	.	.	.
231	2010	CHINA	AEX25348.1	.	.	.	.	.	.	.	.	.	.	.	.	.	.	.	.	.	.	.	.
232	2010	CHINA	AFD61558.1	.	.	.	.	.	.	.	.	.	.	.	.	.	.	.	.	.	.	.	.
233	2010	CHINA	AFP43244.1	.	.	.	.	.	.	.	.	.	V	.	.	.	.	.	.	.	.	.	.
234	2010	FRANCE	CCA61128.1	.	.	.	.	.	.	.	.	.	.	.	.	.	.	.	.	.	.	.	.
235	2010	FRANCE	CCA61129.1	.	.	.	.	.	.	.	.	.	.	.	.	.	.	.	.	.	.	.	.
236	2010	FRANCE	CCA61130.1	.	.	.	.	.	.	.	.	.	V	.	.	.	.	.	.	.	.	.	.
237	2010	FRANCE	CCA61131.1	.	.	.	.	.	.	.	.	S	.	.	.	.	.	.	.	.	.	.	.
238	2010	GABON	AFM38220.1	.	.	.	.	.	.	.	.	.	V	.	.	.	.	.	.	.	.	.	.
239	2010	IND-AP	AEJ18138.1	-	-	-	-	-	-	-	-	-	-	-	-	-	-	-	-	-	-	-	-
240	2010	IND-DEL	AEV42237.1	.	.	.	.	.	.	.	.	.	.	.	.	.	.	.	.	.	.	.	.
241	2010	IND-DEL	AEV42239.1	.	.	.	.	.	.	.	.	.	.	.	.	.	.	.	.	.	.	.	.
242	2010	IND-DEL	AEV42241.1	.	.	.	.	.	.	.	.	.	.	.	.	.	.	.	.	.	.	.	.
243	2010	IND-DEL	AEV42243.1	.	.	.	.	.	.	.	.	.	.	.	.	.	.	.	.	.	.	.	.
244	2010	IND-DEL	AEV42245.1	.	.	.	.	.	.	.	.	.	.	.	.	.	.	.	.	.	.	.	.
245	2010	IND-DEL	AEV42247.1	.	.	.	.	.	.	.	.	.	.	.	.	.	.	.	.	.	.	.	.
246	2010	IND-DEL	AEV42249.1	.	.	.	.	.	.	.	.	.	.	.	.	.	.	.	.	.	.	.	.
247	2010	INDIA	AEE60792.1	.	.	.	.	.	.	.	.	.	.	.	.	.	.	.	.	.	.	.	.
248	2010	INDIA	AEE60793.1	.	.	.	.	.	.	.	.	.	.	.	.	.	.	.	.	.	.	.	.

249	2010	IND-KA	AEX92848.1	.	.	.	.	.	.	.	.	.	.	.	.	.	.	.	.	.	.	.	.
250	2010	IND-KA	AEX92849.1	.	.	.	.	.	.	.	.	.	.	.	.	.	.	.	.	.	.	.	.
251	2010	IND-ORISSA	AFA41483.1	.	.	.	.	.	.	.	.	.	V	.	.	.	.	.	.	.	.	.	.
252	2010	IND-ORISSA	AFA41484.1	.	.	.	.	.	.	.	.	.	V	.	.	.	.	.	.	.	.	.	.
253	2010	IND-ORISSA	AFA43355.1	.	.	.	.	.	.	.	.	.	V	.	.	.	.	.	.	.	.	.	.
254	2010	IND-ORISSA	AFA43356.1	.	.	.	.	.	.	.	.	.	V	.	.	.	.	.	.	.	.	.	.
255	2010	IND-ORISSA	AFA43357.1	.	.	.	.	.	.	.	.	.	V	.	.	.	.	.	.	.	.	.	.
256	2010	IND-ORISSA	AFA43358.1	.	.	.	.	.	.	.	.	.	V	.	.	.	.	.	.	.	.	.	.
257	2010	IND-ORISSA	AFA43359.1	.	.	.	.	.	.	.	.	.	V	.	.	.	.	.	.	.	.	.	.
258	2010	IND-ORISSA	AFA43360.1	.	.	.	.	.	.	.	.	.	V	.	.	.	.	.	.	.	.	.	.
259	2010	IND-ORISSA	AFA43361.1	.	.	.	.	.	.	.	.	.	V	.	.	.	.	.	.	.	.	.	.
260	2010	IND-ORISSA	AFA43362.1	.	.	.	.	.	.	.	.	.	V	.	.	.	.	.	.	.	.	.	.
261	2010	IND-ORISSA	AFA43363.1	.	.	.	.	.	.	.	.	.	V	.	.	.	.	.	.	.	.	.	.
262	2010	IND-ORISSA	AFA43364.1	.	.	.	.	.	.	.	.	.	V	.	.	.	.	.	.	.	.	.	.
263	2010	IND-ORISSA	AFA43365.1	.	.	.	.	.	.	.	.	.	V	.	.	.	.	.	.	.	.	.	.
264	2010	IND-ORISSA	AFA43366.1	.	.	.	.	.	.	.	.	.	V	.	.	.	.	.	.	.	.	.	.
265	2010	SENEGAL	ADG95919.1	.	.	.	.	.	.	.	.	.	.	.	.	.	.	.	.	.	.	.	.
266	2011	COMBODIA	AFM35612.1	.	.	.	.	.	.	.	.	.	V	.	.	.	.	.	.	.	.	.	.
267	2011	COMBODIA	AFM35614.1	.	.	.	.	.	.	.	.	.	V	.	.	.	.	.	.	.	.	.	.
268	2011	COMBODIA	AFM35616.1	.	.	.	.	.	.	.	.	.	V	.	.	.	.	.	.	.	.	.	.
269	2011	COMBODIA	AFM35618.1	.	.	.	.	.	.	.	.	.	V	.	.	.	.	.	.	.	.	.	.
270	2011	COMBODIA	AFM35620.1	.	.	.	.	.	.	.	.	.	V	.	.	.	.	.	.	.	.	.	.
271	2011	COMBODIA	AFM35622.1	.	.	.	.	.	.	.	.	.	V	.	.	.	.	.	.	.	.	.	.
272	2011	COMBODIA	AFM35624.1	.	.	.	.	.	.	.	.	.	V	.	.	.	.	.	.	.	.	.	.
273	2011	COMBODIA	AFM35626.1	.	.	.	.	.	.	.	.	.	V	.	.	.	.	.	.	.	.	.	.
